# Molecular Crowding: The History and Development of a Scientific Paradigm

**DOI:** 10.1021/acs.chemrev.3c00615

**Published:** 2024-03-11

**Authors:** Caterina Alfano, Yann Fichou, Klaus Huber, Matthias Weiss, Evan Spruijt, Simon Ebbinghaus, Giuseppe De Luca, Maria Agnese Morando, Valeria Vetri, Piero Andrea Temussi, Annalisa Pastore

**Affiliations:** † Structural Biology and Biophysics Unit, Fondazione Ri.MED, 90100 Palermo, Italy; ‡ CNRS, Bordeaux INP, CBMN UMR 5248, IECB, University of Bordeaux, F-33600 Pessac, France; § Department of Chemistry, University of Paderborn, 33098 Paderborn, Germany; ∥ Experimental Physics I, Physics of Living Matter, University of Bayreuth, 95440 Bayreuth, Germany; ⊥ Institute for Molecules and Materials, Radboud University, Heyendaalseweg 135, 6525 AJ Nijmegen, The Netherlands; # Lehrstuhl für Biophysikalische Chemie and Research Center Chemical Sciences and Sustainability, Research Alliance Ruhr, Ruhr-Universität Bochum, 44780 Bochum, Germany; 7 Dipartimento di Scienze e Tecnologie Biologiche, Chimiche e Farmaceutiche, Università degli Studi di Palermo, Viale delle Scienze, 90128 Palermo, Italy; 8 Dipartimento di Fisica e Chimica − Emilio Segrè, Università degli Studi di Palermo, Viale delle Scienze, 90128 Palermo, Italy; 9 Università Federico II, Via Cynthia, 80100 Napoli, Italy; 10 King’s College London, Denmark Hill Campus, SE5 9RT London, United Kingdom

## Abstract

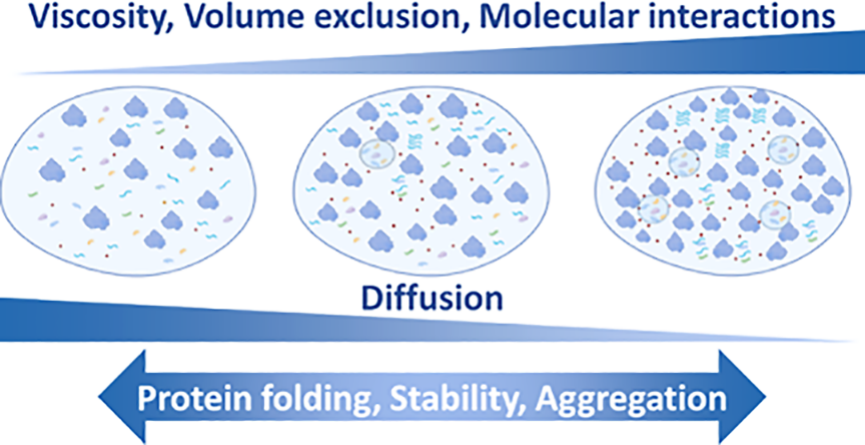

It is now generally accepted that macromolecules do not act in isolation but “live” in a crowded environment, that is, an environment populated by numerous different molecules. The field of molecular crowding has its origins in the far 80s but became accepted only by the end of the 90s. In the present issue, we discuss various aspects that are influenced by crowding and need to consider its effects. This Review is meant as an introduction to the theme and an analysis of the evolution of the crowding concept through time from colloidal and polymer physics to a more biological perspective. We introduce themes that will be more thoroughly treated in other Reviews of the present issue. In our intentions, each Review may stand by itself, but the complete collection has the aspiration to provide different but complementary perspectives to propose a more holistic view of molecular crowding.

## 1 Introduction to the Concept of Molecular Crowding

The great majority of biophysical studies of biological macromolecules are performed in dilute solutions, containing at most a dilute buffer and some salt, in addition to the molecule under study, to have highly controlled conditions. The *in vivo* situation is instead different in many ways. The most obvious difference is the presence of large amounts of different macromolecules. It has been estimated that the fluid inside cells of *E. coli* contains between 300 and 400 g L^–1^ of macromolecules, representing 30–40% of the cell volume.^1^ These values constitute the maximum macromolecular concentration in prokaryotic cells and have been used by virtually all papers dealing with crowding since the introduction of the concept by Allen Minton^2^ and used by Minton himself.^3^ Recently, there have been several new estimates for these concentrations.^4−7^ Model et al.^4^ summarize in a table the data for 15 different organisms and/or cell types. They confirm that the value for *E. coli* is 300–400 g/L, whereas all other values are lower with a minimum of 9 g/L for rat kidney. Illmer et al.^7^ emphasized also the relevance of studying macromolecule concentrations of specific organelles. The blood plasma contains 80 g L^–1^ of proteins. This observation led Allen P. Minton to define the solution inside cells as “crowded”, and to stress the role that excluded volume effects play on protein function and stability.^2,8^ Crowding should not be confused with another concept, that of confinement, which nevertheless is often used interchangeably. Confinement refers to a space limitation and the constraints determined by it. Crowding refers to a more dynamic situation, where molecules are restrained by the presence of many others. The two concepts are certainly related but do not entirely overlap: proteins can for instance be crowded both in an organelle but also in the bloodstream, much as in a very wide space people could group in a specific area. On the other hand, molecules are certainly confined in a small organelle, without necessarily being under crowding conditions.

The main consequence of crowding was attributed by Minton to the exclusion of volume, although the importance of volume exclusion for biomolecules had previously been recognized by Ogston and Laurent already in the 1960s based on studies of the connective tissue polysaccharide hyaluronan.^9^ If we consider macromolecular crowders as hard objects, their sheer presence limits the volume available to other macromolecules, with consequences on the conformation and interactions with other molecules. It is important to notice that a “corollary” of the concept of crowding has been that of assuming that cells, at least the prokaryotic ones, could be considered as “bags full of macromolecules”. In this view, if one evaluates the total number of macromolecules in the cell, the volume of solution accessible to the protein under study is much lower than the volume of the whole cell, simply because only the unoccupied volume can be used. The popularity of the “bag full of macromolecules” model was enhanced by a famous picture published by McGuffee and Elcock^10^ in which the 50 most abundant macromolecules inside a prokaryotic cell were shown in a dynamic molecular model of the bacterial cytoplasm. The image is magnificent but probably misleading, because it gives the (false) impression that all volume inside the cell is occupied by macromolecules. The “bags full of proteins” model is too simplistic, mainly because many macromolecules are part of complexes and thus unable to move inside the cytoplasm. Accordingly, the model was seriously criticized by James Clegg^11^ and Paul Srere^12^ who both regarded the “bag model” seriously doubtful, if not utterly wrong.

Abandoning this model does not imply that crowding is not important, just the opposite. This is also because there are many situations in which the actual concentrations of macromolecules are really very high, particularly when crowding is combined with confinement. Despite its obvious importance, the concept of crowding was not followed for years until Minton published a clear thermodynamic interpretation.^13^ We are now more than 20 years later and the concept is fairly accepted. Many studies have been carried out to explore very different aspects of crowding. The aim of this special issue is precisely that of discussing the very different implications. The present Review wants to be an introduction, by its very nature far from being exhaustive, to a field which is reaching some maturity, even though still much will be needed to be done before it is fully elucidated.

## 2 Playing in the Dark: The Early Models of Molecular Crowding

In this section, we will discuss the history and development of the concept of crowding with the aim of guiding the reader through the complexity of the field and its evolution. We will see that historically the concept of crowding was first conceived by physicists working in the area of polymer physics who treated the problem mostly in terms of entropic contributions. When the concept evolved, enthalpy was taken into consideration.

### 2.1 In the Origin It Was Only Entropy

The first contribution to the idea of molecular crowding does not come from the field of biochemistry/biophysics but from the physics of colloids. The term “colloid” was coined by the British chemist Thomas Graham in 1861 to describe “pseudo-solutions” of particles *dispersed in another liquid, solid, or gas medium*, and characterized by a low rate of diffusion through membranes and a lack of crystallinity.^14^ Examples of colloids could be gels, mayonnaise, or gelatin. In two pioneering contributions, Asakura and Oosawa introduced a purely computational model, named after the authors AO model, in which particles (colloids) in a bath of noninteracting macromolecules experienced an attractive force exclusively of entropic origin.^15,16^ The macromolecules were modeled as permeable spheres corresponding to chains in an ideal or “theta” solvent, that is a solvent in which polymers act as *ideal* chains. Once the distance between the surfaces of two particles dropped below the size of the noninteracting macromolecule, the macromolecules were excluded from the volume between the approaching particles, thereby losing entropy. As a consequence, the zone between the approaching colloids was depleted from macromolecules, leading to the term “depletion interaction” among colloids. The range of the interaction potential was determined by the size of the macromolecules, and its depth increased with the polymer concentration. Although not yet directly applicable to cellular systems, the authors were fully aware of the implications of their work for the field of biophysics including cellular systems, which are crowded by macromolecules.

Independently of the work on colloid–polymer mixtures (CP-mixtures), another line of research evolved that was dealing with purely entropic excluded volume effects, aiming at a better understanding of the process of macromolecular discrimination via size exclusion chromatography.^17^ Ogston calculated the probability for spheres to penetrate into a suspension of rods by treating the mutually excluded volume between the spheres and the rods. The suspension of rods served as a model for the immobile phase of a chromatography process. Giddings et al.^18^ later extended such statistical mechanics calculations to spheres and rods equilibrating between a bulk liquid and an immobile phase, represented by pores of various simple shapes and size distribution. The pores were modeled by a random-pore network created by surfaces with random placement and orientation in space. It is obvious that these dense phases, represented by either a suspension of rods, by pores with size distributions and various shapes, or by random-pore networks established a crowded system with excluded volume effects exchanged with the migrating particles. In 1970, Ogston stressed the analogy of excluded volume interactions between solute particles and pieces of an immobile crowded environment to the excluded volume interactions between solute particles of different kinds with one kind of particle establishing the crowded environment.^19^ He did so by explicitly considering the excluded volume effects among spheres only, among spheres and rods and among rods only. As we will outline below, such efforts did not remain unnoticed by the upcoming community working in the field of biophysics/biochemist as it occurs in living systems like cells and were soon adopted by it.

### 2.2 When Models Meet Experimental Validation

A first experimental validation of the depletion interactions in CP-mixtures was based on the addition of polystyrene chains to a dispersion of polystyrene microgels in toluene, revealing phase separation of a concentrated colloidal suspension.^20^ Further evidence for depletion interactions in CP-mixtures remained scant for the next 20 years. Experimental work only picked up pace when in 1976 Vrij published a model based on binary mixtures of two polymers, of a polymer and a colloid, and of two colloids.^21^ Vrij’s work provided a treatment of different types of binary systems and an explicit consideration of the solvent quality for macromolecular chains under crowded conditions. The author considered different solvent qualities covering the full range from the theta condition in which chains adopt an unperturbed (ideal) chain conformation, to the good solvent limit where the polymer chains are swollen because of the greater affinity to solvent than to other chain segments. Vrij calculated the interaction potentials among the colloids and predicted osmotic second virial coefficients of any selected component. This model was in principle testable by light scattering experiments. Although this contribution exceeded significantly the pioneering results by Asakura and Oosawa,^15,16^ Vrij became aware of the Asakura and Oosawa’s results only after his own publication. De Hek and Vrij reported on CP-mixtures consisting of organophilic silica spheres mixed with polystyrene chains in cyclohexane under theta conditions thereby meeting the model of crowding spheres mutually penetrable in a perfect way.^22,23^ Comparison of these data with the same type of mixtures in toluene, a good solvent for polystyrene, revealed a drop in the amounts of macromolecules required to trigger phase separation by a factor of 3 as compared to the same components in cyclohexane.^24^

Gast and co-workers^25^ were the first to explicitly calculate phase diagrams in which the interaction strength among the colloids was expressed as the concentration of the macromolecular crowder plotted versus the volume fraction of the colloids in coexisting phases. These authors could successfully discriminate between a fluid–fluid and a fluid–solid phase separation, where solid in the latter case meant a long-range order of colloids. To this end, an AO-potential was added to a hard sphere reference potential and the phase behavior was successively calculated via thermodynamic perturbation.^25^ These calculations took into account the variable size of the macromolecules expressed as the radius of gyration (*R*_*g*_) and the radius of the colloidal particles (*R*). The relevance of the size ratio was stressed by demonstrating that a fluid–fluid phase separation in addition to fluid–solid phase separation occurred only at *R*_*g*_/*R* > 0.3, reproducing the trend observed by De Hek and Vrij,^23^ who had reported an increase of the polymer concentration, at which phase separation was triggered as the size of the polymer decreased.

These studies were thus the first to validate theory experimentally and to provide a good integration between experiments and calculations.

### 2.3 Further Contributions of Polymer Science

**Figure 1:**
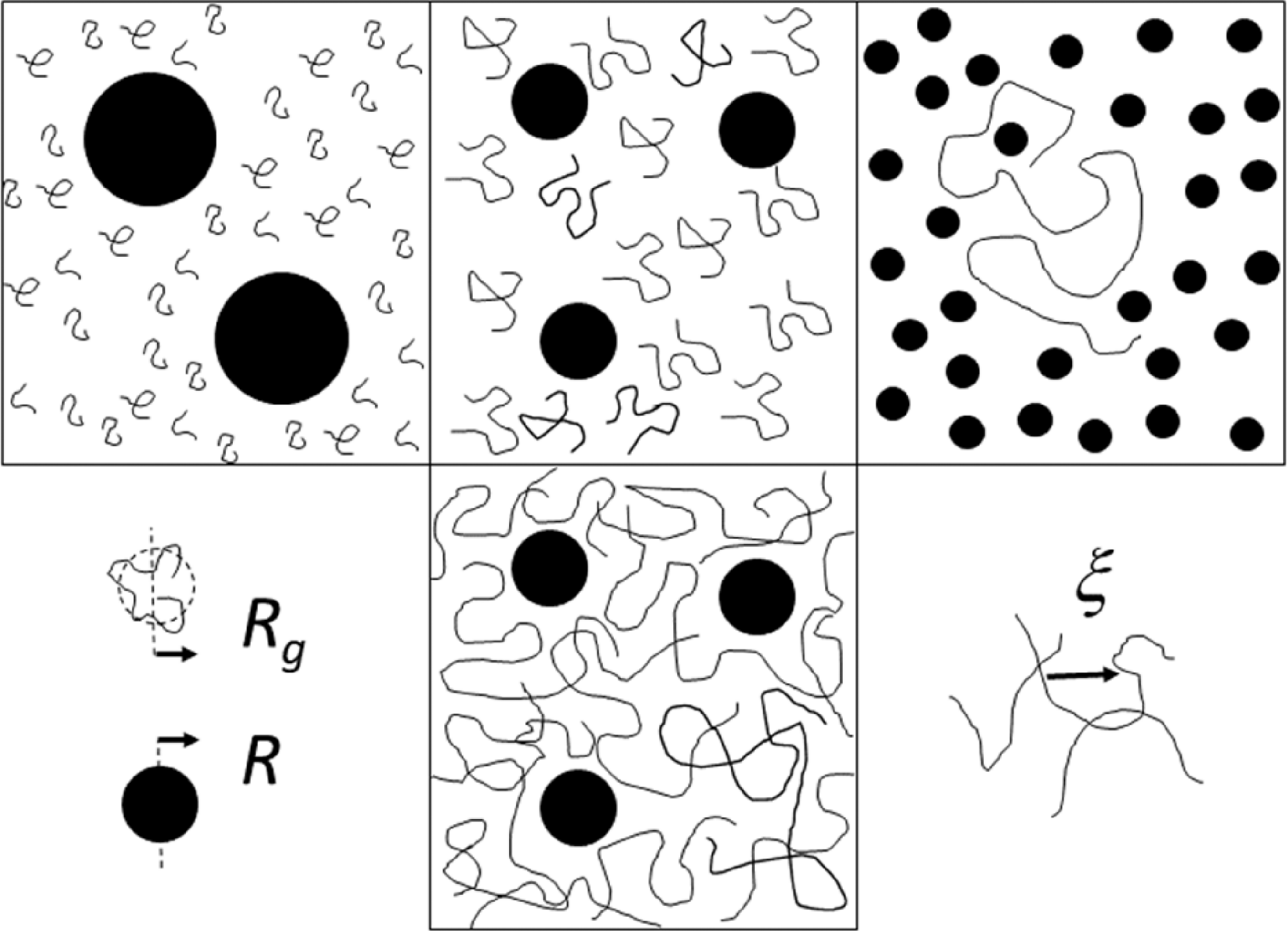
Top: CP-mixture in the colloid limit (*R*_*g*_ < *R*) with macromolecules as crowder (left), *R*_*g*_*∼ R* (center), CP-mixture in the protein limit (*R*_*g*_*> R*) with colloid-like proteins as crowder (right). Bottom: dimensions of colloids and macromolecules (left), colloids in semidilute solution of macromolecules with macromolecules as crowder (center), correlation length ξ (right).

Meanwhile, progress in polymer science had added a further criterion to distinguish different types of macromolecular solutions. Aside from differentiating good solutions from ideal or theta solutions, semidilute polymer solutions were introduced and distinguished from dilute solutions. When increasing the polymer concentration, a regime of semidilute solution is reached once the polymers start to touch and interpenetrate each other. Beyond this crossover, the solution properties do no longer depend on the polymer size expressed as the radius of gyration *R*_*g*_. Since the concentration in terms of monomer density can still be considered as low, the term semidilute solution was coined for such systems (Figure 1).

In the regime of a semidilute solution, the relevant length scale of the polymers *R*_*g*_ turns into a correlation length ξ, which decreases with increasing polymer concentration. For CP-mixtures where the colloidal particles are larger than the correlation length (*R* > ξ), both Joanny et al.^26^ and De Gennes^27^ in 1979 predicted a depletion zone, which decreases with increasing polymer concentration. To discuss the impact of the correlation length on the interaction potential, the authors were forced to discriminate between two regimes, (*R* > ξ) and (*R* < ξ). The scaling law for the minimum of the interaction potential was predicted to be proportional to −(*R*/ξ) for *R* > ξ, whereas it was calculated to −(*R*/ξ)^4/3^ for *R* < ξ.^26,27^

Prior to 1992, theoretical approaches^15,16,22,23,25^ did not consider that partitioning of the macromolecular components may occur among two separating phases differing in the concentration of the colloidal particles. Thus, the interaction potential among colloids was assumed the same in both phases. It was only with Lekkerkerker et al.^28^ that such partitioning was observed by calculating phase diagrams of CP-mixtures using a new theoretical approach based on a mean-field approximation to estimate the free volume available to macromolecules within a colloidal suspension. The approach was thus given the name Free Volume Theory (FVT). The free volume fraction was estimated by combining the Widom’s particle insertion method^29^ with the scaled particle theory by Reiss et al.^30^ Similar to the findings by Gast et al.,^25^ fluid–fluid phase separation in addition to fluid–solid phase separation occurred for *R*_*g*_/*R* > 0.3. In analogy with atomic systems, the fluid phase with lower colloid concentration was considered to represent the “gaseous” state of the colloids. The phase with higher colloid concentration established the “liquid” state of the colloids. In agreement with these predictions, Ilett et al.^31^ reported the coexistence of a triple point and a critical point capping liquid–liquid phase separation (LLPS) in the phase diagram by means of poly(methyl methacrylate) spheres suspended in solutions of polystyrene chains under theta conditions.

It is worth mentioning that the theoretical approaches^15,16,22,23,25,28^ introduced above treated the macromolecular component only implicitly, considering it a modulator of the interaction potential between colloids or planar plates immersed as a pseudo single-component in a solvent. As correctly pointed out in a critical Review by Zukoski and co-workers^32^ in 2002 that treated the entropically driven phase behavior, the theoretical approaches on CP-mixtures published by Gast et al.^25^ and Lekkerkerker et al.^28^ are more appropriate for macromolecules under theta conditions. The validity of these approximations decreases as the size ratio *R*_*g*_/*R* increases and exceeds 1, and as the concentration of macromolecules exceeds the overlap concentration. The relevance of semidilute solutions was fully acknowledged already at that time.^26,27^ The work by Zukoski and co-workers^32^ impressed by presenting a highly systematic study of silica colloids in the presence of polystyrene chains under good solvent conditions at variable size ratio within the range 0.026 ≤ *R*_*g*_/*R* ≤ 1.395, based on five different polymer samples. Using a polymer concentration normalized by the respective overlap concentration as the ordinate of a phase diagram, the authors recovered a retreat of the spinodal line of LLPS to higher volume fractions of colloids with increasing the size of the polymeric crowder, i.e., with increasing the size asymmetry ratio, in contradiction with theoretical predictions based on the models of Gast et al.^25^ and Lekkerkerker et al.^28^

The trend observed by Zukoski and co-workers^32^ could be adequately reproduced by a new theoretical approach by Fuchs and Schweitzer,^33,34^ based on an analytical polymer reference interaction site model (PRISM). Later on, another approach developed by the Dutch school of colloid and polymer science turned out to be similarly successful as the PRISM-Ansatz.^35−38^ The authors extended the FVT by explicitly considering excluded volume interactions also among the crowding polymer chains by means of renormalization group theory, and succeeded in satisfactorily predicting the phase behavior of CP-mixtures at variable size asymmetry ratios and solvent qualities of the polymer component.

These early studies demonstrate how much the field of crowding was originally inspired by polymer physics. This knowledge can help us to understand where some concepts come from and how they have evolved with time.

### 2.4 Introducing Complexity

Full appreciation of the relevance of the asymmetry size ratio by polymer physicists eventually led to a distinction of two limiting cases of CP-mixtures.^39^ The CP-mixtures discussed so far predominantly included colloids with size values larger than the macromolecules, which act as crowder. Such cases were termed the “colloid limit” (Figure 1). Inspired by features like the compaction of DNA chains in cells, an opposing limit of CP-mixtures was identified, where large macromolecules are exposed to small colloidal particles with *R*_*g*_/*R* ≫ 1. Under these conditions, colloid-like globular proteins were assumed as crowders, and the condition was given the term “protein-limit”. Growing attention to this limit paralleled the breath-taking progress made in the biochemistry and biophysics of living systems. Structured proteins undergo folding processes during or soon after their synthesis on the ribosome, leading to the more compact native state. Such conformational changes of polymer chains occur in the cellular crowded environment and are expected to heavily respond to variations of these crowded conditions. Early studies in the protein-limit considered the phase behavior at semidilute solutions of large macromolecules in the presence of small hard spheres and noticed the absence of phase separation.^27,40,41^ It was only Van der Schoot who considered the conformation of large macromolecules in the presence of small colloidal particles acting as crowders. He predicted a collapse of the dilute macromolecular chains in the presence of sufficiently large amounts of small colloids which represent the crowding proteins.^42^ In this model, the entropy gain anticipated by the small colloids forced large chains into more compact structures. This feature could for the first time be verified experimentally with large polystyrene chains under good solvent conditions in the presence of synthetic small colloids,^43,44^ and later on with polyethylene glycol (PEG) in water in the presence of Ficoll 70 as a crowder.^45^ A considerable collapse of polystyrene chains in toluene as well as of PEG chains in water was noticed as the crowder content increased. Small angle neutron scattering experiments (SANS) with both systems were particularly powerful as they facilitated contrast matching of the colloidal crowder and the solvent.^43−45^

Although still simplified, these further studies added an important layer of complexity that became later on beneficial for studies which were considering the problem from a different point of view as we will see in the next paragraphs.

### 2.5 Adopting a Different Perspective

We have until now adopted the perspective of polymer science. However, this should not prevent us from directing our attention toward the evolution of a new research focus, which gradually turned into the field of macromolecular crowding. Two pioneering publications by Laurent illustrate well the origin of this new research focus.^9,46^ The author initially investigated the solubility of various proteins in the presence of dextran to confirm an excluded volume effect of the crowder on protein solubility.^9^ Later on, he turned his focus to the question of how macromolecular crowding affects enzyme catalysis.^46^ In his 1971 publication, he expressed the relevance of such studies for biological processes by stating “*The reason for studying enzyme reactions in polymer media may not be immediately obvious. It represents, however, an initial attempt to describe the environment in which intracellular enzymes function.*” In this statement, the author was realizing that while most biophysical studies are carried out in dilute solutions where only the directly involved reactants are considered, the reality requires a more complex situation.

Minton and co-workers made further progress in the field of macromolecular crowding not only by publishing numerous experimental studies but also by introducing a new theoretical approach.^8,13^ The polymer physics community used the interaction potential among colloids as modified by polymer-mediated depletion interactions to describe the phase behavior of CP-mixtures, and attempted to analyze how small colloidal particles affect the conformation of large polymers. Minton suggested instead to analyze cellular processes by correcting the activities of all directly participating reactants. These corrections were based on excluded volume effects caused by sufficiently inert crowders, which inevitably decreased the free volume available to the reactants, and thus increased their activities accordingly. These excluded volume effects were accounted for by the nonideal part of the chemical potential of the reactant according to the equation:
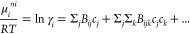
with the logarithmic activity coefficient of component i being expanded in powers of concentrations *c*_*j*_ and *c*_*k*_ of crowders *j*, *k*, and with *B*_*ij*_ and *B*_*ij*k_ the binary and ternary cluster integrals, determined by the mutually excluded volume.^47^ Closed form expressions for ln γ_*i*_ were calculated with the help of scaled-particle theory for hard spherical particles.^30^ An alternative route to estimate ln γ_*i*_ involved direct calculation of the molar covolumes (the *B*_*ij*_ coefficients) as done for sphere–sphere or sphere-rod encountered by Ogston.^19^

Like the approaches adopted by polymer and colloid scientists, Minton’s approach was based on purely entropic effects. Minton applied this concept to various cellular processes, classifying them into three types: (i) homogeneous enzymatic catalysis,^8^ (ii) conformational changes of biomacromolecules,^48^ and (iii) protein self-assembly,^49^ with the latter leading to hierarchical structures with specific tasks or to the formation of amyloid aggregates. Clearly, these three types of processes do not include the LLPS observed with CP-mixtures (sections 2.2 and 2.3) as a consequence of depletion interactions among the colloids.

As discussed later on (section 6.4), LLPS generating membraneless droplets with high concentrations of proteins has to be added as a fourth class of cellular processes. Frequently, the main participants of such droplet formation are intrinsically disordered proteins, which are flexible and thus more polymer-like than compact globular proteins. This type of phase separation should be distinguished from the phase separation of colloids in CP-mixtures, which relies on attractive interactions mediated by depletion of macromolecules. In the case of LLPS of single component macromolecular solutions, purely hard sphere interactions among monomeric units being of purely entropic origin are superimposed by net attractive interactions among the monomeric units. This was first predicted by Flory^50^ and Huggins^51−53^ and has now become common knowledge in polymer science supported by compelling experimental evidence. The Flory–Huggins theory was thus an early attempt to bring enthalpic interactions into polymeric systems. These studies emphasized the need to consider enthalpic interactions on top of purely entropic depletion effects.

## 3 Enthalpy versus Entropy

Crowding agents are often described as “inert or non-interacting macromolecules” that are part of the “environment” of a reaction or a biological process. This description conveys the false impression that they are mere bystanders that do not partake in any process. On the contrary, crowder agents are very active agents as we will discuss in the next paragraphs. Following the Minton’s original formulation,^2,8,13^ several studies attributed the influence of crowding essentially to entropic effects. Subsequently, other authors, for instance Miklos et al.,^54^ analyzed the effects of crowding from a new point of view: they drew attention to the fact that studies on macromolecular crowding often had ignored chemical interactions between the crowder and the test protein. This concept was not new: in 1973, Anfinsen had in fact drawn attention to the role that weak surface contacts play in protein chemistry.^55^ In 1983, McConkey coined the term quinary structure to define the interactions between a protein and the rest of the intracellular environment, as a sort of additional step beyond primary, secondary, tertiary and quaternary structures.^56^ More recently, the Pielak laboratory has pioneered the relaunching of the concept as we shall see in the next paragraphs.^57,58^ The importance of quinary interactions has now been completely accepted by the scientific community and is considered an important element to understand a number of observations, among which the absence of the nuclear magnetic resonance (NMR) spectrum in some of the *in-cell* studies in prokaryotes.^59,60^

### 3.1 Crowders as Deceiving Bystanders: Toward a More Thorough Perspective

In a study on chymotrypsin inhibitor 2 (CI2), Miklos et al.^54^ showed that the presence of the crowder poly(vinylpyrrolidone) may stabilize the test protein by soft interactions with the native state of the protein. This was an important turning point in the concept of crowding. Most studies had assumed, at least implicitly, that only volume excluded by the presence of macromolecular crowders was acting on protein stability. In reality, it is almost impossible to find crowders completely “inert”, as it will be discussed later on. It is relatively easy to find crowders that do not interfere with electrostatic forces but very difficult to find crowders that do not form nonbonding weak forces with the molecule under study.

In fact, our understanding of the complexity of protein stabilization in crowded solutions evolved when the Pielak laboratory explicitly hinted at the entropy/enthalpy antinomy. Wang et al.^61^ stated that crowding can affect protein stability in two ways: by hard-core repulsions or by soft chemical interactions. In a study by NMR on ubiquitin based on amide proton exchange, these authors found that the contribution of chemical interactions is substantial and, in many cases, larger than the contribution from simple repulsions. The possible balance between entropic repulsions and enthalpic contributions was summarized by Sarkar et al.^62^ in a thorough review of different studies. These authors reached the conclusion that the large number of soft interactions between a crowder and the protein under study can overcome the stabilizing steric effect coming from excluded volume even if they are nonspecific and weak.

The explicit contraposition of entropy and enthalpy in the effect of crowders on protein stability was accepted also by other authors. For example, Senske et al.,^63^ when studying the thermal unfolding of ubiquitin, observed that addition of other solutes (glucose, dextran, PEG, guanidinium, and urea) led to both enthalpic and entropic destabilization. The authors argued that the classification of cosolute effects based on their excess enthalpic contributions results in a comprehensive thermodynamic model.

However, when comparing entropic and enthalpic effects in crowding, there might have been some confusion between understanding the influence of crowding and reproducing the environment in the cell, i.e., performing a cell mimic. The essential difference between “crowding” and “in-cell mimic” was well described in a recent paper, in which the authors concluded that the combination of lysis buffer and Ficoll could be a simple but effective new *in vitro* mimic of the intracellular environment to study protein folding and stability.^64^

The difficulty of finding experimental support for entropic stabilization of proteins is complex, but the main reason is that the contribution of forces different from entropic ones has been underestimated. One study in which it was attempted to measure the extent of enthalpic interactions of the crowders directly was performed by Alfano et al.^65^ who measured the presence of direct interactions between crowders and the protein under study by NMR spectroscopy, a technique very sensitive to the effects of specific interactions on chemical shifts. These authors showed that specific (enthalpic) interactions of Yfh1 protein with several synthetic crowders are minimal. Thus, in this case, the influence of crowders on protein stability could be attributed almost entirely to entropic factors.

Finally, it has been argued that both synthetic and physiologically relevant crowders pose challenges not seen in dilute solution experiments, such as increased solution viscosity, high background, and decreased signal quality due to interactions between crowders and test proteins.^66,67^ However, in our opinion, these effects are an inherent component of the effects of crowding and as such cannot be considered unwanted phenomena, although they of course contribute to adding new layers of complexity to our understanding of crowding.

### 3.2 Crowding Environments

The most obvious reason to study macromolecular crowding effects on biomolecular systems is to understand how they behave in the crowded space of a living cell, packed by up to 400 mg of macromolecules per ml of cytosol, or in other biological fluids.^1^ As most biochemical assays and analytical tools are carried out in aqueous buffer solutions, the effect of the cellular environment is rarely taken into account when such experiments are used to interpret *in vivo* function or dysfunction.

However, as previously discussed, the cell is not only a crowded bag of molecules. One example that illustrates this fact is protein stability (not referring to stability in terms of the degradation of the protein). *In-cell* NMR spectroscopy revealed that ubiquitin is destabilized in cells,^68^ whereas the B1 domain of protein G (GB1) is stabilized in the cytoplasm of *E. coli* as compared to aqueous solution.^69^ Site-specific mutations of a truncated version of superoxide dismutase have different impact on the protein stability in dilute aqueous solution and in cell.^70^ Osmotic perturbations that lead to changes in crowding density,^71−74^ cell stress^75^ or differentiation,^76^ change protein stability in different ways.

Such differences are the consequence of the complexity of the cellular environment subdivided into distinct compartments resulting in a multiscale heterogeneity. Membrane-encompassed^77^ and membraneless^78,79^ organelles enrich and modify protein folding stability in different ways as compared to the cytoplasm. Compartments vary in their chemical makeup^80^ and pH gradients exist even within the cytoplasm itself.^81^ Driven by a myriad of cosolute interactions, entropic and enthalpic effects modulate the folding free energy landscape of a protein in different ways, specific to the local environment and the nature of the protein itself, as well as the state of the cell, e.g., in healthy and disease conditions.

In addition to crowding and cosolute interactions, biological processing such as post-translational modifications must be considered explicitly when interpreting *in-cell* effects in comparison to the test tube. For protein stability, molecular chaperone interactions have a significant impact, destabilizing proteins by preferentially binding to the unfolded state (i.e., by having a holdase function).^75^ The amplitude of destabilization depends on several factors, among which the local enrichment and the activity of the chaperones.^78,82^

**Figure 2:**
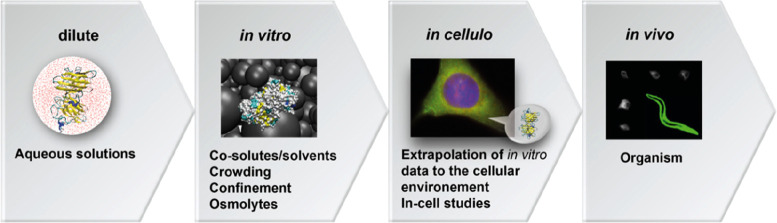
Workflow to understand biomolecular reactions in biological environments with increasing complexity.

Finally, a large cell-to-cell variability in terms of crowding effects is expected in multicellular model organisms such as in zebrafish.^83^ In the cited paper, the authors suggested that *in-cell* (crowding) experiments should be validated by a workflow that rigorously compares *in vitro* the different contributions of crowding, cosolutes and biomolecular interactions, to lead to a comprehensive interpretation of the results obtained at the cellular level and in multicellular model organisms (Figure 2). This important lesson should be probably kept in mind by all authors working directly *in vivo* without the support of *in vitro* data.

### 3.3 In Search of the “Perfect” Crowder

Models are the bread and butter of scientists, even more so if physicists or chemical physicists. Thus, several models have been developed to study macromolecular crowding under controlled conditions, following two different and in some way opposite philosophies. According to the first school of thought, crowders should be “inert” molecules, that is those that do not form interactions with the molecule under study. A large number of studies have, for instance, used on purpose “inert” polymers, such as PEG, dextran, Ficoll, and poly(sodium 4-styrenesulfonate) (PSS).^84^ These polymers are often available as polydisperse species, thus having a distribution of molecular masses. The idea behind this choice has mainly been the attempt of separating the entropic contribution from the enthalpic one, and be able to discriminate the two effects. However, the principle sounds simple, but it is difficult to put it into practice: some of the thought-to-be inert crowders can in fact interact with proteins, although with weak and nonspecific interactions.^59^ In support of this statement is the work by Lee et al.^85^ who tried to elucidate the structural bases of the PEG/protein recognition by solving the structures of complexes of PEG with the Fabs of two anti-PEG antibodies by X-ray diffraction. The authors could not find any common pattern in the interactions in the two structures, as expected for complexes determined by weak nonspecific interactions.

The complexity of the topic is also well illustrated by a study by Kozer et al.^86^ who studied the interaction in a range of concentrations, from dilute to semidilute to concentrated solutions. The authors monitored the association of two proteins, TEM1-β-lactamase and the β-lactamase inhibitor protein, in solutions containing crowding agents of different molecular weights, from monomers (ethylene glycol, glycerol, or sucrose) to polymers like PEG of different molecular weights (from 0.2 to 8 kDa) and Ficoll. In all solutions, it was found an inverse linear relation between the translational diffusion of the proteins and viscosity, in general agreement with the Stokes–Einstein relation. Deviations of the association rates from the Stokes–Einstein equation were related to the three distinct regimes of polymer concentrations: in the diluted regime, PEGs interfere with protein association by introducing a repulsive force originated from preferential hydration. In the semidiluted regime, it is possible to observe faster association rates due to the depletion interaction, which causes an attraction between the two proteins. At high concentrations of crowder, PEGs slow down the association between, as a function of their concentration.

It is also important to notice that, in most experimental studies on the influence of crowders on protein stability, it is employed only one crowder at very high concentrations. Under these conditions, even very weak enthalpic interactions become effective. The situation in the cell is different because different enthalpic interactions can be averaged out by interactions with different macromolecules.

In the second perspective, scientists have instead tried to reconstitute the cellular environment as more accurately as possible and thus used proteins or mixtures of proteins, implicitly or explicitly accepting a mixture of effects as, under these conditions, enthalpic effects are possible if not likely. The simplest models adopt single proteins, usually chosen among those known to have a low tendency to interact. Examples of these proteins are the bovine pancreatic trypsin inhibitor (BPTI), ribonuclease A, lysozyme, β-lactoglobulin, hemoglobin, and bovine serum albumin (BSA).^87−91^ Studies of mixed crowders were also conducted, and the advantages of mixed crowding over homogeneous crowding were independently suggested by different groups.^92−94^ Zhou tested, for instance, the effects of mixed crowding on protein stability and suggested that optimal crowding effects could be obtained by adjusting mixing ratios between crowders’ populations.^92^ Shah et al. also suggested a role for enthalpic interactions in mixtures using an *in silico* approach at lower than physiological temperatures (27 °C).^95^ A more systematic and thorough study was carried out by Dewavrin et al.^96^ who demonstrated that the crowding efficiency yielded by homogeneous crowders is far below the situation observed *in vivo*, where the physiological microenvironment contains heterogeneous populations of crowders. The authors convincingly showed, using the kinetics of collagen nucleation and fiber growth, that mixing crowders of different sizes (polyvinylpyrrolidone 20 (PVP), dextran, and Ficoll) generates a synergistic effect: small crowders bring about extra volume occupancy when in the vicinity of bigger crowders, beyond the volume occupied by their structure. Molecular simulations also showed that the volume excluded in a crowder mixture is significantly higher than the added volumes of single crowding agents.

Along the line of heterogeneous crowders, but tackling a different level of complexity, more complex alternatives were proposed. The Pastore group, for instance, introduced the use of hen egg white as a simple natural medium, which offers most of the characteristics of the media of crowded cells, that could be used by any researcher without difficulty and inexpensively, despite some inherent limitations discussed in the original paper.^97^ The authors showed that hen egg white does not affect the fold or stability of proteins, but modulates the dynamics and can increase dramatically the aggregation kinetics of proteins with an inbuilt tendency to associate. This effect was partly explained by an excluded volume effect and partly by interactions with other proteins from the milieu.^98^

Other groups, such as the Pielak laboratory, have had a different brilliant solution and used lyophilized *E. coli* lysates or cellular extracts which in principle contain a plenitude of different components to mimic the cytoplasm.^62,99^ This model is powerful and attractive but, as all models, has its own limitations: lysates usually do not contain lipid membrane components, so critical surface–tracer interactions may be absent. As *in ovo*, lysates also include a large number of unidentified and uncharacterized proteins that might interact specifically or nonspecifically with the probe molecules in a noncontrollable way. Finally, the preparation of lysate is likely to disrupt naturally occurring microcompartmentation and distort or eliminate spatial distributions and local compositions of macromolecules present within the intact cell. As an alternative, Good and colleagues reviewed the use of confined *Xenopus* cytoplasmic extracts as models of intracellular environments providing compelling reasons for its usage.^100^ The extracts may be confined within emulsified microdroplets whose size may be controlled by microfluidic techniques or layered atop a supported lipid bilayer within a flow channel. Unfortunately, the use of this promising system has been relatively limited, probably because it requires the availability of *Xenopus* eggs.

These studies show that several different models have been developed offering interesting possibilities of mimicking the complexity of the cellular environment, also without necessarily working *in vivo*. The choice of the most suitable one will certainly be related to the specific case and can thus not be decided *a priori*.

### 3.4 Crowders versus Solvation: The Golden Ratio

The question of how the crowder size affects processes in cells is straight and simple but leads to complex answers. Complexity already comes into play when considering simple systems governed by entropic effects only. According to the approach by Minton,^2,8,101^ this can be considered by using the volume excluded by the crowder to the probe species, thus establishing a free volume accessible to the probe species. This accessible volume does not only depend on the size of the crowder but also on the size of the probe particles. According to Minton “*On geometric grounds one would not expect crowding by large macromolecules to greatly affect the behavior of small molecules or significantly smaller macromolecules, which can more easily fit into interstices between large molecules*”.^102^ This is a clear comment that grasps the nature of the difference in size between molecules. However, the significantly smaller components are probably more appropriately described as cosolvents rather than crowders as in an elegant contribution by the Pielak lab.^103^

Complexity is further illustrated with simple CP-mixtures as they were introduced in section 2. CP-mixtures include systems with the size of the macromolecules (*R*_*g*_) smaller than the colloid radius (*R*) whereby the macromolecules are acting as crowder, which are predominantly used to analyze the phase behavior of colloidal suspensions. In the opposite size limit which assumes the colloids as much smaller than the macromolecules, the resulting systems serve to investigate how colloids, now acting as the crowder, affect the size of much larger macromolecules. This perspective adds complexity to the relevance of crowder size (or size asymmetry ratio) in crowding.

For some of the classes of processes typically occurring in cellular environments, a first insight into the simple question of the impact of the size asymmetry ratio is provided both by theory as well as by experimental evidence. It has to be stressed, however, that experiments in this field require availability of model polymers and macromolecules, with both components acting either as probe or crowder, at variable and well-defined molar mass values.

The first process to be briefly addressed is the phase separation of colloidal particles triggered by a macromolecular crowder. The influence of the crowder size is particularly significant below the overlap concentration of polymers, with the most striking feature corresponding to the width of the attractive potential among the colloids proportionally increasing with increasing macromolecular size. Above the overlap concentration, which decreases with increasing crowder size, any further impact of the crowder size is of minor relevance. In two highly systematic studies, Zukoski and cow. investigated the influence of the crowder size on the phase behavior of colloidal particles for the crowding macromolecules under good solvent and theta conditions.^32,104^ Easily accessible standard samples of polystyrene at variable molar mass values served as macromolecular crowders. As already mentioned, macromolecular crowders under good solvent conditions shift the spinodal for the LLPS to higher colloidal volume fractions with increasing crowder size if the crowder concentration is normalized by the respective overlap concentration.^32^ The same experiments with macromolecules under theta conditions revealed the reversed trend.^104^

An impact of the crowder size is less obvious and less well investigated when looking at the conformational changes of macromolecules as the probe particles in the presence of a crowder, which in CP-mixtures is represented by small hard sphere colloids. According to an atomistic model by Qin and Zhou, the free energy difference between the transformation of a denatured Cytochrome *b*_562_ to native Cytochrome *b*_562_ in the absence and in the presence of crowders is negative implying that macromolecular crowding favors the more compact native state.^105^ This preference for the native state under crowding conditions increases with the decreasing size of the crowder. In the cited study, the crowder was equal to or larger than the probe molecule Cytochrome *b*_562_. The same trend was observed in Wang–Landau simulations published by Taylor and co-workers on CP mixtures covering size values of the crowder from the size of a monomer to the size of the collapsed polymer,^106^ and by computer simulations by Scolari et al.,^107^ which extends to a size of the colloids to a regime even smaller than the length of a monomeric unit of the macromolecules. The authors revealed an increase of the temperature for the coil-to-globule transition with decreasing crowder size with the effect gradually disappearing as the crowder size approached the size of the native protein. These theoretical predictions were complemented by an experimental study based on single molecule spectroscopy to measure the size of probe protein molecules in the presence of PEG as a macromolecular crowder. Soranno and co-workers presented a highly systematic study on the influence of PEG covering a degree of polymerization 1 < *P* < 500 on the size of four different proteins all belonging to the family of intrinsically disordered ones.^108^ The authors found a compaction of all four proteins upon increase of PEG content, and, at a given PEG content, a gradual compaction of the proteins with increasing size of the PEG molecules. The latter results, which covered a broader range of crowder sizes, are in contrast to the trend observed in computer simulations.^106,107^ However, both simulation techniques applied hard sphere crowders,^105,106^ whereas Soranno and co-workers used flexible macromolecules as crowders, stressing the relevance not only of the size asymmetry ratio but equally important, of the nature of the crowder.^108^

In 2017, Alfano et al. addressed the question of whether and what is the optimal size of crowders.^65^ Using yeast frataxin, a protein that undergoes cold denaturation above zero degrees under quasi-physiological conditions,^109^ allowing accurate determination of *ΔC*_*p*_, the authors explored the effect of crowders of different sizes, and showed that protein stability would be affected by volume exclusion with a more pronounced effect when the crowder volume is closer to that of the protein under study. The study was carefully designed to rule out the role of soft interactions as supported by NMR evidence. The enormous differential effect of PEG on cold denaturation was explained in terms of a variation in water activity, which goes according to Privalov’s interpretation of cold denaturation.^110^ More recently, other authors have considered the same question and concluded that to maximize the crowding effect, the crowding agent and the protein should have a similar size. When the crowder is too small, as it is the case of cosolvents, water and any small molecule, we would rather call it a solvation effect rather than crowding. Conversely, molecular crowding is referred to as molecular confinement rather than crowding when the molecular weight of the macromolecular cosolutes increases to the point that they can be considered effective immobile obstacles forming a lattice with pores that can be occupied by the molecular species of interest.

Another important process is the self-assembly of proteins in the absence and presence of crowding. Self-assembly, if following a monomer addition process, can be treated as a polymerization with growing ends also termed living polymerization or chain growth. The equilibrium constant of such a living polymerization exhibits close analogy to a precipitation of the polymers or aggregates since the equilibrium constant *K* = 1/[M]_e_, with [M]_e_ being the concentration of monomers in equilibrium with the aggregates, or the solubility limit of a precipitate. Such a self-assembly is simply promoted by the decreasing volume accessible to the monomers, which for a given crowder decreases with the increasing size of the monomer and accordingly with the size of the crowder.^102^ An excellent example, where the influence of the size of a macromolecular crowder on self-assembly of a protein has been analyzed was published by Fink and co-workers.^111^ The authors looked at the fibrillation of α-synuclein in the presence of PEG at four different molar mass values of the polymer and succeeded to demonstrate that at a given crowder concentration fibrillation was the more accelerated the larger the polymer molar mass became.

In conclusion, we have analyzed in detail the relevance of the nature and size of the crowder and how these parameters influence the observed effects.

## 4 Techniques to Study Crowding

A thorough discussion on the subject of the standing and emerging techniques suitable for the study of molecular crowding may be covered in a different chapter of the present special issue. It is however helpful at this stage to reflect on a number of general problems. By the very nature of the problem that we want to study, molecular crowding must be tackled by techniques that are able to discriminate a specific reporter within a mesh of other molecules. This requirement rules out many (but not all) the spectroscopies, like for instance circular dichroism spectroscopy because there would be no ways to distinguish between crowder and protein under study.

An obvious approach is to use labels to selectively investigate conformations and conformational transitions of the reporter. Fluorescence microscopy is a highly sensitive technique for this purpose with well-established protocols for site-specific labeling *in-cell* and *in vitro*. In combination with other methods like Fast Relaxation Imaging, it is possible to analyze the kinetic and thermodynamic signatures of crowding effects with high spatial resolution.^112^ Similarly, NMR and EPR spectroscopy have proven very useful to study protein structure and stability under crowding conditions,^113−115^ as well as during phase transitions.^98,116^ Under this perspective, a recent Review discusses in detail how the combination of fluorescent-based and NMR experiments can be even more effective to explain protein homeostasis in terms of structure and stability.^117^

It is also possible to analyze single reporter molecules within living individual eukaryotic cell.^118^ Alternatively, reporters can be labeled using NMR-sensitive or EPR-sensitive probes or isotopes yielding information on the local chemical environment, conformation or populations of the probe.^119^ Reporters that specifically reveal crowding effects in living cells need to undergo well-defined conformational transitions under changing crowding densities. As such they need to be calibrated *in vitro* under different crowding conditions (using different types and concentrations of crowders) and need to be insensitive to other environmental factors such as pH or ionic strength, at least within the physiologically relevant range. As an example, FRET-labeled PEG (which is often used as a crowder itself) was used as a sensor to study molecular compression induced by excluded volume in living cells.^73^ Boersma et al. designed a spring-like protein backbone labeled terminally by genetically encoded fluorescent proteins.^72^ Such experiments showed the broad diversity of crowding effects including excluded volume effects and intermolecular interactions in cells that led to different net outcomes of the crowding effect, depending on the biomolecular probe but also on the cellular environment or the cellular conditions.

Common to these *in-cell* techniques is that labeled reporters need to be transferred into the cellular environment (e.g., by microinjection or electroporation) or be genetically encoded. Both methods bear the risk of inadequate localization or concentration. Depending on the fluorescence method used, it is often nontrivial to show that the labels do not interfere with the read-out of the reporter.

Complementary to crowding-reporter experiments are high-resolution and label- and reporter-free cryogenic structural techniques. For example, rapidly developing methods in cryo-electron tomography allow to reach a resolution of 4 Å and beyond.^120,121^ Such methods report accurately on the macromolecular density of specific subcellular environments. Finally, it is important to mention the new opportunities created by the fourth generation of synchrotron facilities, based on the multibend achromat lattice concept and able to surpass the brightness and coherence attained by previous synchrotrons.^122^ The combination of static and dynamic light scattering through the second virial coefficient,^123,124^ and small-angle X-ray scattering^125−128^ is a particularly powerful approach to describe interaction between proteins for dense systems. Neutron and X-ray based methods such as neutron spin echo (NSE) or X-ray Correlation Spectroscopy (XPCS) are also complementary approaches ideally suited for *in-cell* studies as they are capable of characterizing diffusive processes over atomic distances. Small Angle Neutron Scattering (SANS) offers the opportunity of contrast matching, which is particularly useful in the structural analysis of components in a complex environment as it occurs in model systems for crowded solutions. Whereas deuteration in principle allows to amplify the scattering contrast of a single component, variation of the solvent composition by changing the ratio of deuterated and hydrogenated solvent molecules may lead to full contrast matching of all but one component, thus enabling scientists to address the morphology of a single component in the mixture. The latter technique had been successfully applied to investigate the structure of macromolecules in a crowded (but invisible) environment.^43−45^ Techniques such as single-molecule force spectroscopy, fluorescence-based assays, and advanced imaging methods can also provide valuable insights into the kinetics, thermodynamics, and spatial organization of intermolecular interactions in crowded environments.^129−132^ However, challenges exist in accurately interpreting the experimental data due to the complexity of the crowded environment and the multitude of factors influencing it.

Finally, future research in the field of crowding will certainly benefit greatly from the integration of a multidisciplinary approach in which the collaboration between biochemists, physicists, and biologists may foster a comprehensive understanding of this intricate system at different resolution scales.

## 5 Effect of Crowding on Stability and Dynamics

We shall now examine the effect of crowding on very different aspects of the cellular organization and functioning. The effect of crowding on proteins has widely been explored and it is well recognized that it can affect molecular diffusion,^133−136^ dynamics,^137−141^ interactions,^133,141−147^ and stability both of proteins and nucleic acids.^65,113,148−150^ In the coming section, we will cover some of these aspects, although the field is so complex that attempts to capture its full complexity into model systems must be deemed to do it still only partially.

### 5.1 Crowding and Protein Stability

The native structure of a protein corresponds to an ordered or disordered state (local or global) which is closely connected to its function or dysfunction.^151−154^ Many but not all proteins spontaneously reach stable functional globular structures via the “folding process”. This leads to the formation of native intramolecular contacts which stabilize a specific three-dimensional arrangement. Some proteins adopt their functional conformation only after the encounter and binding to their physiological partner.

**Figure 3:**
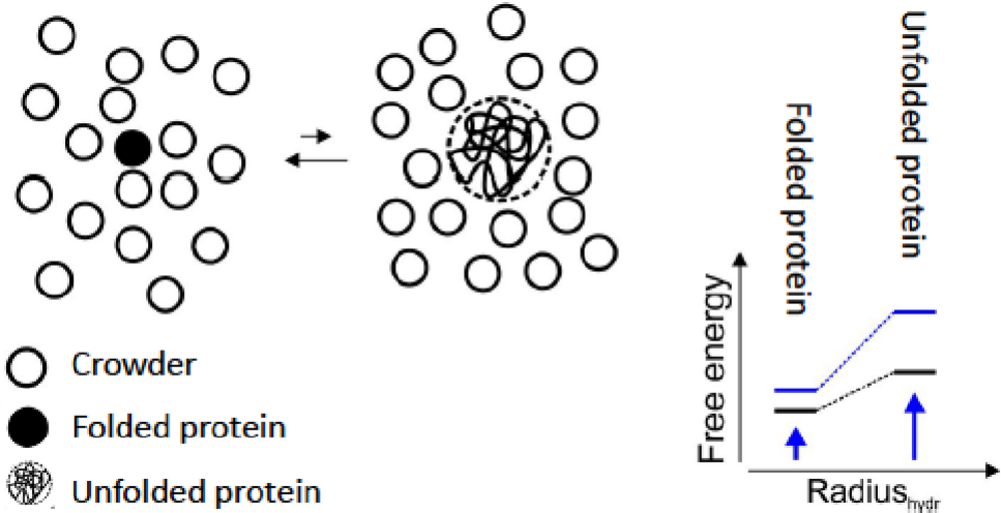
Excluded volume effect favors the more compact conformations of proteins due to hard-core repulsions. The folded native state of a protein is favored over the expanded denatured state because of its compact structure. The excluded volume effect increases the free energy of both, the folded and the unfolded state. However, the increase in free energy is larger for the expanded unfolded state leading to an overall stabilization of the native protein.

The stability and the folding/unfolding of a protein can be described in terms of free energy variations where the enthalpy term takes into account the “binding energy” (electrostatic interactions, hydrogen bonding, and van der Waals interactions), while the hydrophobic interactions are described by entropy-driven processes. The consequence that captured most attention in early studies of molecular crowding was the prediction of the influence of volume exclusion on protein thermal stability. In a paper by Minton, the unfolded protein was described as a sphere with a radius corresponding to the radius of gyration, that is experimentally measurable.^13^ On the basis of this model, Minton predicted increases of the transition midpoint temperature in the range of 5–20 °C. Formulated in a slightly different way, we could say that the excluded volume effect may shift the equilibrium folded-to-unfolded states toward the folded one. The effect can be understood thinking that, in a crowded environment, the expanded unfolded state is disfavored as compared to the more compact folded state, because of the excluded volume effect (Figure 3).

Many theoretical contributions following the original theory formulated by Minton and followers and purely based on entropic grounds tried to justify large increases in protein stability, as measured by a large increase in melting temperatures in temperature unfolding studies. However, most of the experimental studies measuring the thermal stability under crowded solutions showed only a modest increase of the unfolding temperature^155^ or a mild stabilization of the folded structure in crowded environments.^54,105,156,157^

To justify these discrepancies, different explanations were suggested.^158^ One was that the shape of the unfolded state used by Minton^13^ was too simplistic because the assumption that the unfolded form of any protein is spherical is largely inaccurate.^159^ More elongated conformations of the unfolded state would still be consistent with radii of gyration described in several SAXS studies and would agree with a modest increase in the volume of unfolded states with respect to that of the folded state.^159^ A different important explanation was formulated by the Pielak laboratory,^54,61,63,160−164^ who suggested that enthalpic effects, coming from weak or quinary interactions with the crowder, were at least as relevant as entropic effects in determining protein stability in crowded solutions. This implies that the measured increase of stability would be the average of two potentially conflicting contributions which might lead to an overall decrease in protein stability. Along these lines, Wang et al. highlighted and compared the entropic and enthalpic contributions of crowding to the stability of ubiquitin showing how crowding effects depend on temperature.^61^ The authors showed that molecular crowding has a destabilizing effect at low temperature, while at higher temperature it has a stabilizing effect. The threshold temperature depends on the nature of the crowder, being higher for protein crowders, with respect to polymer crowders. Chu et al. observed by NMR how different crowding agents affected protein folding at the individual residue level, stabilizing more effectively either regions of the protein structure that are prone to local unfolding, or the unfolding of the global structure.^156^

**Figure 4:**
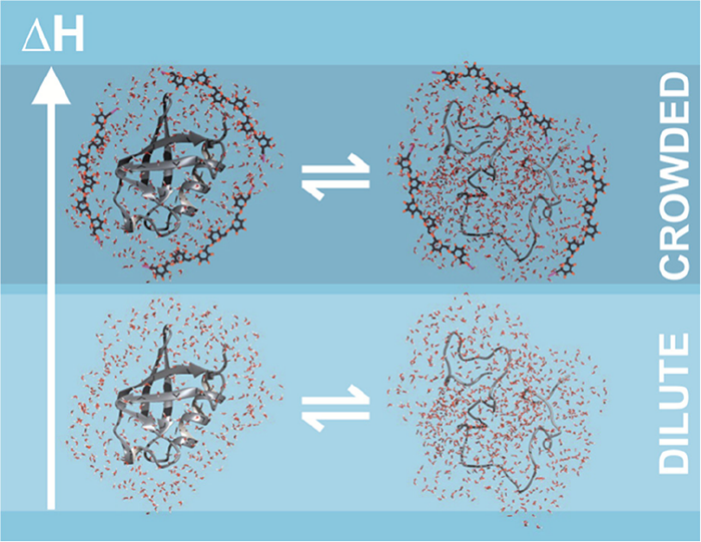
Folding equilibrium of ubiquitin in dilute and crowded solution. The native state of ubiquitin is stabilized relative to the denatured state via an enthalpic mechanism.

Finally, it is important to mention that molecular crowding affects the hydration shell around protein molecules, particularly if the crowder is strongly excluded from the protein surface.^165,166^ This can have dramatic effects on folding and aggregation processes as solvent mediated interactions are critical in these phenomena.^167^ Indeed, the hydration water shell works as a structural and dynamical connection but also as an active constraint. Crowding acts on the hydration shell by significantly reducing water mobility and self-diffusion.^168,169^ In parallel, crowding also reduces dielectric response, altering the energy landscape.^168^ Enhancement of electrostatic interactions results in strengthening hydrogen bonding between proteins and water molecules, thus resulting in fold stabilization (Figure 4).^170,171^ On the other hand, water ordering reduces the entropic benefit of isolating hydrophobic residues from the solvent, favoring partially unfolded states.^172^ At high crowder concentrations, the properties of hydration water change significantly from those of bulk solvent. This may change protein–protein interactions which water mediates by providing an extensive and highly dynamic network of hydrogen bonds.

We may thus conclude that the forces that stabilize a protein can be strongly modulated by weak interactions with the environment.

### 5.2 Diffusion, Dynamics, and Trafficking in Crowded Environments

In addition to its impact on steady-state properties, crowding has also a strong effect on the diffusion and transport of macromolecules. In the following, we will focus on three-dimensional bulk systems and soluble macromolecules. For a comprehensive review on crowded membrane systems, we refer the reader to Guigas et al.^173^ Starting again from the simplest approximation, namely that crowders are just inert spheres, some basic consequences of crowding on transport can be drawn from colloidal science as mentioned above. Early work by Einstein for dilute suspensions predicted the effective viscosity to increase with the colloidal volume fraction as η = η_0_(1 + 2.5φ), where φ is the volume fraction of the spheres, and η_0_ is the viscosity of the suspending medium (g/cm s).^174,175^ Meanwhile, measurements and theory have extended this first estimate, revealing an up to 100-fold increase in viscosity in crowded colloidal suspensions below the glass transition (i.e., at φ ≈ 0.58).^176^ Therefore, the diffusion constant *D* ∼ 1/η of a tracer can be expected to be significantly reduced in crowded media if its size is similar to that of the crowders. Beyond the simple approximation of inert spheres, one needs to take into account the polymer-like nature of crowders like PEG, dextran, nucleic acids, or proteins. Semidilute polymer solutions and polymer melts show a rich rheological phenomenology, such as the emergence of viscoelasticity, even when charges and specific interactions are neglected. Therefore, it can be expected that crowded fluids exhibit nontrivial material properties that affect transport and diffusion. In line with this notion, strongly reduced mobilities, i.e., up to 10-fold lower diffusion constants have been observed for proteins and tracer particles in intracellular fluids and cell extracts.^177^ More striking, however, was and is the emergence of an anomalous diffusion in crowded media, i.e., a nonlinear growth of the mean square displacement (MSD) over several time scales. This phenomenon has been observed with a variety of techniques in many systems, from the cytoplasm of living cells^178−181^ to biomimetic crowded solutions.^182−184^

The observed anomalous diffusion often boils down to a sublinear power-law scaling of the MSD, i.e., <*r*^2^(*t*)> ∼t^α^ with α < 1. Several advanced theoretical models can be used to rationalize this scaling^185,186^ and a large toolbox of observables allows comparison between experimental data with these models.^187^ In the context of crowded media, two generic models have been particularly useful in interpreting experimental data: Obstructed diffusion (OD) and fractional Brownian motion (FBM). The OD model assumes that crowders form a static and self-similar confinement on the time scales of interest, e.g., a static percolation cluster of impenetrable obstacles that is reminiscent of an archipelago.^188^ A tracer will move freely in the residual fractal space of this confining maze, yielding a sublinear scaling of the MSD with α ≈ 0.55 in three dimensions.^189,190^ However, if the obstacles are made mobile, the anomaly will gradually subside, and normal diffusion (α = 1) will eventually be regained.^191^ Unlike the fairly static OD model, FBM incorporates the dynamic facets of crowding because it is a mathematically sound model for a random walk of tracers in a viscoelastic fluid, such as semidilute polymer solutions. Therefore, in FBM the MSD exponent 0 < α ≤ 1 reflects the relative impact of the elasticity-driven memory (which enforces an antipersistent motion) as compared to the memory-devoid dissipative viscosity. In essence, tracers that undergo an antipersistent random walk of the FBM type move similar to the saying “two steps forward one step back”. FBM has been seen experimentally in many crowded systems, e.g., for inert tracers in the cytoplasm^180,181^ or in biomimetic fluids.^184^

In general, the size of the crowder relative to the tracer is a key parameter that determines whether slower or even anomalous diffusion will emerge. Yet, knowing the hydrodynamic radius of a protein alone may not be sufficient. For example, intrinsically disordered proteins (IDPs) are typically less affected in their diffusion than globular proteins of the same hydrodynamic radius.^140^ This can be understood by considering that a polymeric tracer (such as IDPs) can still move in a reptation-like fashion through crowded media in which globular tracers are already trapped. Moreover, biochemical interactions between crowders and proteins can update all of the above, adding yet another layer of complexity.

Changes in protein diffusion naturally influence the protein activity by modifying collision and association rates,^133^ and crowding-induced anomalous diffusion can even cause significant changes in pattern formation.^192^ Besides these transport aspects, crowding also affects the local dynamics of macromolecules. Crowding has been reported to alter the equilibrium between the open and closed conformations of DNA hairpin structures^193^ and several proteins,^194^ pushing the systems toward more compact closed states.

The dynamics of IDPs was also shown to be greatly influenced by crowding. NMR spectroscopy showed that both backbone and site-chain dynamics are influenced site-specifically,^195^ leading to increased friction coefficients.^196^ Again the effect on IDPs was shown to be crowder-specific, although in general a compaction of the IDPs was observed with crowding.^108,141^ The degree of compaction is also crowder-size dependent and could be quantitively explained by modeling IDPs as polymers rather than as globular proteins.^108^ König and co-workers tested the effect of different cellular environment on an IDP,^140^ showing that the eukaryotic Hela and HEK cells have a much lower crowding content than bacteria, resulting in very little effect on IDPs. Similarly, in-cell NMR and EPR studies have shown that the IDP α-synuclein remains highly dynamic and disordered in eukaryotic cell models.^197,198^ Along the same lines, Popovic et al. demonstrated that the in-cell NMR spectrum of the yeast protein Yfh1 is invisible, with the only exception for the highly flexible N-terminus.^199^

Altogether, this brief overview highlights that crowders are not mere bystanders also from the dynamic point of view. Rather, transport and structural dynamics are modulated in nontrivial ways.

### 5.3 Crowding and Nucleic Acid Structure

Although much attention has been paid to proteins, it is worth also considering the effects of crowding on nucleic acids since this subject seems to be a topic of increasing interest. The mechanisms by which crowding influences the structure and stability of RNA and DNA resemble those acting on the previously discussed protein systems, with excluded volume effects by macromolecular crowding leading to entropic stabilization.^200−203^ However, the two polynucleotides retain their own peculiarities. Due to the complexity of the subject we will refer the reader to a recent review by Singh and co-workers for a discussion about double-stranded DNA.^204^ We will instead briefly discuss RNA and single-stranded DNA whose exquisite flexibility proposes specificities that are not observed in globular proteins.

In duplexes and hairpins, interactions may play a particularly important role given that the uniform negative charge of the RNA backbone at the exterior and their hydrophobic interior make RNA particularly susceptible to interactions with a variety of polar and nonpolar molecules. However, the effect is not uniform. In an *in vitro* UV study,^205^ it was shown for instance, that high molecular weight PEGs (PEG 4000/8000) have stabilizing effects on the folding cooperativity of a tRNA under physiologically concentrations of Mg2+ (0.5–2 mM) and K+ (140 mM) and in the presence of ∼20% PEG or dextran, whereas the much smaller PEG 200 does not have appreciable effects. On the contrary, low-molecular-weight cosolutes had varying effects on tRNA folding cooperativity, increasing or decreasing it depending on the cosolute.

Other studies demonstrated that interactions with crowders and other cytosolic components (including for example RNA-binding proteins) lead to destabilization and decreases in water activity upon crowding.^206−208^ In an elegant study of the folding stability of a hairpin-structured RNA inside live mammalian cells,^204^ the authors observed that the RNA stability is comparable to that in dilute physiological buffer. On the contrary, the addition *in vitro* of artificial crowding agents, with the exception of high-molecular-weight PEG, led to destabilization caused by soft interactions with the crowder. The authors also showed that RNA stability is highly variable within cell populations as well as within subcellular regions of the cytosol and nucleus. They thus concluded that inside the cell RNA is subject to (localized) stabilizing and destabilizing effects which on average result to an only marginal effect as compared to a diluted buffer.

The presence of crowders seems anyway to influence the compactness of RNA. A small-angle X-ray scattering study was reported on a 64 kDa bacterial group I ribozyme in the presence of PEG-1000, a molecular crowder with an average molecular weight of 1000 Da.^201^ It was shown that PEG favors more compact RNA structures as observed through detecting the transition from an unfolded to a more compact folded state which occurs at lower MgCl_2_ concentrations. The radius of gyration of the unfolded RNA decreased from 76 to 64 Å as the PEG concentration was increased from 0 to 20 wt %/vol.

More recently, quite some work has been carried out to investigate the effect of crowding on the conformation of the G-quartets. These are guanine-rich DNA/RNA sequences that can fold into four-stranded, noncanonical secondary structures composed of stacks of guanines.^209^ Many studies, among which we will cite only a few for lack of space, have concentrated on telomeric G-quartets because these often adopt a mixture of conformations in mutual equilibrium. All authors found that the equilibrium is affected by the crowder (in most cases PEG) concentration and the presence of K^+^ or Na^+^. A study by Heddy and Phan considered, for instance, telomeric G-quartets which can fold into parallel, antiparallel, and (3 + 1) hybrid-1/-2 structures, under the control of the cation present.^210^ It was found that the conformation of a telomeric G-quartet in K^+^ solutions was significantly affected by the presence of PEG 200, shifting the equilibrium between species from a hybrid to a parallel structure. No changes were found in the presence of Na^+^.^211^ Li et al. showed that the structure of the human telomeric G-quartets varies with increasing concentrations of PEG, leading to a structural compaction and increased thermodynamic stability.^212^ A study of the telomeric sequence dG4T4G4 from *Trichoderma aculeatus* in the presence of 100 mM Na+ and PEG 300, propanetriol, or positively charged butylenediamine, pentanediamine, and spermidine reported a conformational transition in the G-quartet from an antiparallel structure to a parallel one.^213^

In addition to shifting the equilibrium between G-quartet conformations, crowding has also an overall stabilizing effect as compared to dilute solution conditions, although the entity of the effect is different for different G-quartets. The melting temperature (*T*_m_) of the human telomeric G-quartet in K^+^ dilute solutions is for instance 68.4 °C, whereas it increases to >80 °C in the presence of 40% (w/v) PEG 200.^211^ A more modest increase of the *T*_m_ from 54.1 to 58.7 °C was observed for the antiparallel G-quartet formed by thrombin aptamer sequences in 40% PEG 200 (w/v).^166^ A close correlation between the G-quartet stability and the molecular weight of the molecular crowder has been observed: for example, PEG 8000 stabilizes the M2 G-quartet (dTAGGGACGGGCGGGCAGGG) to a greater extent than ethylene glycols at 20% (w/v). The effect is comparable for 20% (w/v) ethylene glycols and 10% (w/v) PEG 8000.^214^ The selective behavior of molecularly crowded environments was found to depend on the number of G-tetrad layers. PEG 200 has been reported to stabilize RNA G4s with three and four G-quartets but not those with two G-quartets.^215^

Much more could be discussed on the effect of crowding on nucleic acids, but we have limited our analysis to a few paradigmatic examples that can give an idea on some significant aspects of the topics.

## 6 Effect of Crowding on Phase Transitions

In addition to having effects on the structure and stability of molecules, crowding can strongly modulate phase transitions. Two types of biologically important phase transitions occurring in the cell are the liquid solid transition, that produces molecular aggregates in a usually irreversible fashion, and liquid–liquid phase separation, resulting in the reversible formation of biomolecular condensates. We will discuss both effects in the following sections.

### 6.1 Effect of Crowding on Aggregation and Amyloid Formation

Cells have evolved to have highly controlled environments in which proteins are stable, preventing misfolding/unfolding processes. However, such a complex process may be prone to errors, giving rise to partially unfolded or misfolded proteins and possibly to aggregation phenomena. Independent evidence shows that proteins may sacrifice contacts observed in their native state, favoring intermolecular contacts with other proteins. In this situation, the aggregated states may result more thermodynamically favorable than the native state. Excluded volume effects, for instance, favor self-assembly due to the smaller excluded volume exerted by the fibrillar structures as compared to the respective monomeric building blocks. Aggregation can thus be considered a competing pathway to normal folding.^153,216,217^

**Figure 5:**
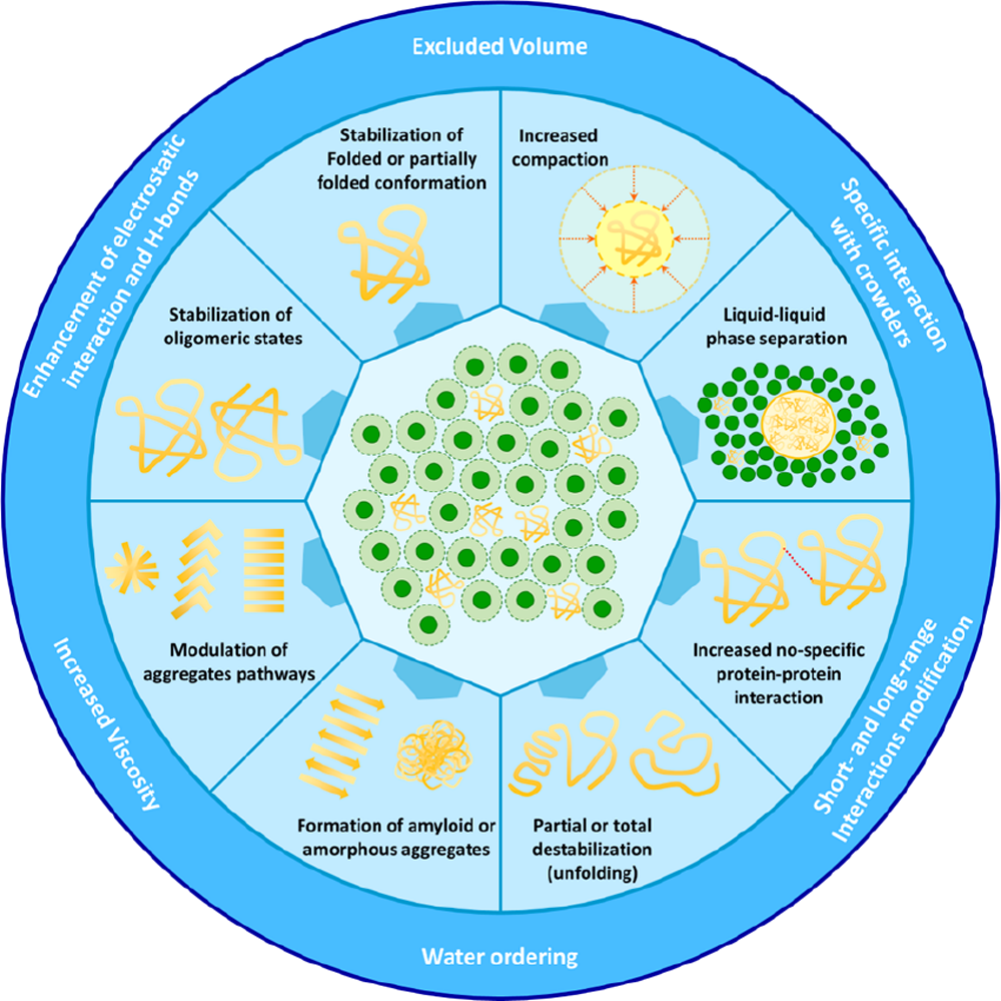
Diagram summarizes the effects of molecular crowding on protein stability and/or on protein ability to interact giving rise to association phenomena leading to different supramolecular structures. Protein aggregation is a complex, often hierarchical, multistep process determined by the interconnection and modulation of multiple mechanisms appearing in different time and length scales. The presence of crowders may impact protein aggregation at different levels involving a complex interplay of various effects, such as excluded volume, changes in solution viscosity, modification of dominant short- and long-range interactions and water ordering.

The influence of crowding on self-assembly and aggregation has been extensively studied because of its pivotal role in various diseases. Experiments in crowded environments have highlighted that protein aggregation is critically different from the same process under dilute conditions. Different independent mechanisms are at play (Figure 5), as we shall see.

The presence of crowding agents may dramatically alter the aggregation pathway and the subtle balance between concurring interactions, highlighting how crowding may drive partitioning between multiple aggregation pathways, determining thermodynamically favorable conformation, helping the system to eliminate the less favorable ones.^218,219^

A shift from different protein states, e.g., from monomeric to oligomeric species, may be dramatic for the onset and the evolution of protein association phenomena and particularly relevant for proteins prone to aggregation. When excluded volume effects dominate and the native state of a globular protein is stabilized, one may expect that supramolecular assembly is disfavored. This is not always the case, as molecular crowding was shown to favor or accelerate globular protein aggregation.^93,220,221^

Hatters et al.^222^ reported how molecular crowding promotes amyloid aggregation for human apolipoprotein C–II, highlighting the effects of volume exclusion. Their results showed that the aggregation pathway was not altered but fastened. Intriguingly, as amyloid formation is a multistage process, it was suggested that crowding may promote or inhibit fibril formation depending on changes in the excluded volume occurring during different stages of the process.^223^ This is accelerated if proteins take up less excluded volumes once in the aggregated state, resulting in an entropy driven lowering of the energy barrier of fibrillar state compared to a crowder-free environment. On the other hand, if the excluded volume increases during the process the rate of aggregation decreases.

Moreover, specific crowding agents may give rise to non-neglectable catalyzing surface-effects. During the formation of insulin amyloid spherulites, the conformation of polysorbate 80 and its ability to form micelles was, for instance, found to modify in a concentration dependent way not only the aggregation process but also the size, the secondary structure and the morphology of the final species.^224^ In 1999, Dobson and co-workers showed that refolding of oxidized lysozyme was not affected by crowding, whereas correct refolding of the reduced protein was antagonized by aggregation at high concentrations of crowding agents. These results showed how crowding could affect protein refolding through competing with proper disulfide formation.^89^

It remains difficult to disentangle the different contributions that modulate cellular aggregation pathways considering also further cellular processes such as chaperone interactions or protein degradation. A study of the effects of chemical and macromolecular chaperones on the aggregation of the islet amyloid polypeptide (IAPP) showed ambiguous effects caused by the intricate aggregation mechanism of the peptide and significant enthalpic contributions.^225^ To reduce complexity, reporter systems specifically sensitive to crowding effects were sometimes used. The well-characterized molecule pseudoisocyanine chloride (PIC) aggregates into fibrillar structures leading to the formation of highly fluorescent J-aggregates.^226,227^ The advantage of using PIC as a cellular sensor is that it is cell-permeable and can be readily used to study cells under different conditions even in multicellular model organisms like the *C. elegans. In vitro* studies revealed that aggregation is promoted by macromolecular crowding agents such as the polysaccharide Ficoll 400 but not by its monomeric building block sucrose.^228^ Under crowding conditions, PIC aggregates at concentrations well below those needed in a buffer solution.

Boersma et al.^229^ developed an intermolecular FRET method with both the donor and acceptor fluorescent proteins at the same reporter protein, in their study of mutant Huntingtin exon1. The construct can be transfected and shows a high FRET read-out upon self-assembly in the living cell. However, as expected these sensors primarily show the engagement of different cellular factors like molecular chaperones on the aggregation state and the self-assembly kinetics. The presence of crowders seems thus to impact protein aggregation through much more than excluded volume effects. It involves a complex interplay of various effects, such as changes in solution viscosity and modification of dominant short- and long-range interactions, that drive the process. These effects can act differentially on the various stages of aggregation, from the protein monomer to the intermediate oligomers and the mature fibers.

Macromolecular crowding increases the effective protein concentration and the solution viscosity, reducing the diffusion rate.^230^ In a simplistic description, the balance between these two parameters may either reduce or increase the aggregation rates. Several studies have reported that macromolecular crowding may induce the stabilization of compact protein conformation, or promote the partial ordering of disordered proteins and, as discussed below, may induce phase transitions^231,232^ or promote nucleation events.^218^ Other effects are local variation of pH and, in general, a reduction of the protein–solvent interactions. Additionally, short- and long-range interactions between proteins and crowding agents should be considered, as they can either have stabilizing or destabilizing effects.

Depending on the specific protein, the solution conditions and the type of crowding agent,^92,219,230,233−237^ the supramolecular assembly can be favored^111,238^ or disfavored^239−241^ resulting in variations of the rate and the pathway of aggregation (e.g., nucleation events or changes in protein conformation, specific molecular interactions), and/or of the nature of intermediate and final species.^242^

Different polymeric crowders are expected to have variable effects on protein aggregation depending on their specific physicochemical properties (e.g., charge, hydrophobicity, size). It is likely that hydrophilic polymers like PEG, dextran and Ficoll act mainly via excluded volume effect while other polymers, proteins or their mix may have specific effects on aggregation.^240^ These conclusions are supported by different studies. The effect of the crowding agents was for instance evaluated on β2m amyloid formation.^243^ In this study, the authors demonstrated that fibril formation was not affected significantly by PEG, whereas was inhibited in the presence of a protein crowder (serum albumin) because of formation of weak interactions between b2m and serum albumin, which stabilize the folded state. Two effects were reported: an increase of the lag phase of aggregation, probably due to increased viscosity, and an increase of fibril amount, due to excluded volume effects. This is interesting because it contrasts with what was observed for other proteins, even if an extreme increase in viscosity is known to decrease the aggregation rates.^111^

Acceleration of amyloid formation of α-synuclein and in particular a reduction of the lag phase was described in the presence of high concentrations of both charged and neutral polymers (proteins, polysaccharides and PEG).^244^ The effect depends on the crowder concentration and the physicochemical features of the protein leading to the hypothesis that excluded volume effects are dominant in favoring association together with decreased solubility of the protein. Other studies reported that each individual step in the aggregation pathway including secondary nucleation may be affected, or that the aggregation may follow an alternative pathway.^245−248^

Results from Breydo et al. showed that in the presence of hydrophilic crowders such as dextran, the formation of amyloid fibrils can be both accelerated or inhibited, depending on the nature of the protein under study.^219^ The authors used different proteins with various degrees of intrinsic disorder and in different oligomeric states. They found that fibril formation is inhibited with proteins that are either already present as stable oligomers or can easily form stable oligomers during aggregation. Conversely, fibril formation of intrinsically disordered proteins is accelerated. Between these two extremes, proteins that have a defined secondary structure and a stable three-dimensional monomeric structure experience an intermediate effect, leading to either mild acceleration or inhibition of aggregation. The authors also suggested that the flexibility of the molecular crowder may play a role in leading to different aggregation pathways: flexible crowders (e.g., dextran) were found to act primarily by excluded volume effects, while more rigid crowders (e.g., hydroxypropyl cellulose) were found to modify the aggregation mechanisms via increased viscosity effect and nonspecific protein-crowder interactions.

This discussion clarifies the difficulty to draw general conclusions on the effects of crowding on protein aggregation and amyloid formation but, at the same time, provides an overview of the elements to keep in mind in trying to predict the crowding effect.

### 6.2 Crowding and Aggregate Polymorphism

Aggregates can be amorphous or amyloids, the latter being characterized by a common highly organized hydrogen-bonded structure which confers high thermodynamic stability. Amyloid aggregates can have different morphologies and sizes, from elongated fibrils and dense microparticles (particulates) to core–shell structures (spherulites), and exhibit a common cross-β-sheet structure.^249^ They can be formed from a lateral arrangement of protofilaments, which exhibit differences at each structural level, i.e., in side chain packing, hydrogen bond networks, as well as secondary and tertiary structure.

Polymorphism of amyloid aggregates has emerged as a key property closely related to pathology. Thanks to recent advances in cryoEM, it was shown for several systems that the conformation adopted by proteins within the amyloid assemblies is disease-specific. Most markedly is maybe the protein tau, involved in a class of diseases called tauopathies that includes Alzheimer’s disease. The amyloid aggregates of tau can take very different structures in different tauopathies, while their structural states seem to be homogeneous within one disease.^250^ The rules dictating this structural diversity remain elusive as it remains to date challenging to reproduce disease-associated structures with recombinant tau isoforms.^251−253^ A similar polymorphism has been observed for other disease-associated proteins such as Aβ,^254−256^ α-synuclein,^257,258^ β2-microglobulin,^259^ amyloid A,^260,261^ Light Chain amyloid,^262,263^ and IAPP.^264−266^

Polymorphisms originate from multiple coexisting aggregation pathways where early intermediates, typically misfolded proteins and/or abnormal IDP conformations, can lead to different amyloid fold. The kinetic and thermodynamic competition between these different pathways may determine which pathways will predominate and will determine the final folds. Although the thermodynamics (i.e., the stability of a given structure) should be the main selection factor for polymorphisms, a computational study by Pellarin and co-workers showed that folds that are energetically less favorable can nucleate faster and therefore become predominant.^267^ Similarly, electron paramagnetic resonance (EPR) demonstrated that the fracture and growth rates determine the final population of tau fibrils.^268^ In other words, the final population can be kinetically selected.

The effect of crowding on amyloid polymorphism is expected to be complex by acting on different processes (Figure 5) although it has not been systematically studied. Complex crowded media such as the human serum were for instance shown to modulate IAPP polymorphism.^266^ A direct mechanism of action of crowders can be to favor the growth of a particular polymorph,^245,269^ favoring a specific amyloid structure. It was also shown by Munishkina and co-workers that crowding agents can stabilize specific intermediates and therefore specific pathways.^218^

Crowders could also stimulate protein–protein interactions, which in turn could favor particular assembly conformations. For instance, Radovan and co-workers showed that inhibition of hydrophobic interactions by high hydrostatic pressure modulates the morphology of IAPP fibrils.^265^ Similarly, crowding has a strong effect on protein hydration by promoting protein partial dehydration, while protein hydration was shown to be a major regulator of amyloid formation.^167,270,271^ It was indeed experimentally demonstrated that crowders affect polymorphism through the perturbation of protein solvation for α-synuclein^272^ and TDP-43.^273^

Finally, a few studies have been carried out on functional amyloids, where polymorphism can be expected to be less important as it does not result from a misfolding event but it is part of the normal function of the protein.^274^ Nevertheless, the work by Siri and co-workers showed that inducing crowding with alginate, a natural exopolysaccharide, modulates the morphology of fapC amyloids, a functional protein involved in biofilm formation.^275^

Thus, the effect of crowding on the specific morphology of the aggregates seems a promising direction to explore in the future, as it could be directly related to a number of important diseases and provide the key for understanding fundamental cellular aspects.

### 6.3 Crowding, LLPS, and Biomolecular Condensates

Among the many fields where the importance of crowding was recently underlined, one of the most recent and prominent one is that where LLPS plays a central role. Crowding not only affects protein stability and aggregation, but also weaker protein–protein and protein-nucleic acid associations that can result in LLPS and the formation of biomolecular condensates (BMCs), also known as membraneless organelles. The formation of these organelles is well distinct, in principle, from protein aggregation, which involves a liquid–solid phase transition. As opposed to aggregation that is usually irreversible, LLPS is a reversible phenomenon.^276−279^ As a result, it can give rise to the entropically driven formation of intrinsically disordered fluids, thanks to the release of water molecules and ions.

Cellular function heavily relies on compartmentalization, where specific biochemical reactions and processes occur within defined spaces. BMCs, such as P granules,^280^ stress granules,^281^ and nucleoli,^282^ are critical players in this process, facilitating spatial and temporal organization of biomolecules without the need for membrane boundaries. BMCs exhibit unique characteristics, including spherical shapes, selective compartmentalization of biomolecules, and high mass-exchange rates with the outer milieu.

**Figure 6:**
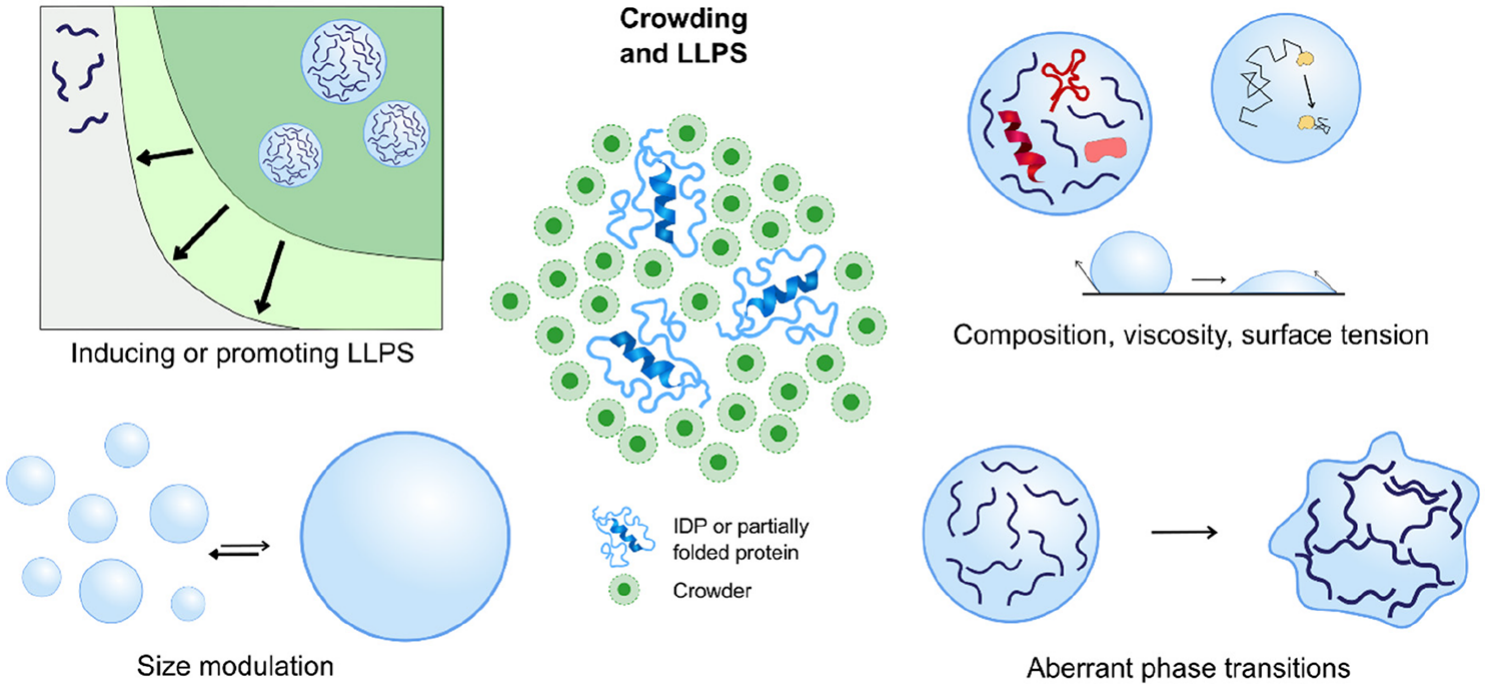
Schematic overview of the effects of molecular crowding (center, green spheres) on LLPS and biomolecular condensates formed by partially disordered proteins (center, blue). Crowding generally promotes LLPS, which is quantified by a shift of the LLPS phase boundary, and the addition of crowding agents can induce LLPS. Crowding agents may also change physicochemical properties of BMCs after formation, including composition (via partitioning), density, viscosity and surface tension (wetting). The presence of crowders can influence the size distribution of BMCs via altered nucleation and coalescence rates. Finally, crowders can induce or speed up aging of BMCs, which may result in kinetically arrested gel-like states and aberrant phase transitions.

At the same time, cellular environments are highly crowded, and this can significantly impact BMC formation and structure. Crowding can induce LLPS by strengthening intermolecular interactions, in a similar way as in protein aggregation. Crowders can thus influence the generation of denser BMCs and can alter condensate composition. Moreover, connected to the thermodynamic reversibility and rapid molecular exchange dynamics of BMCs and at variance with protein aggregation, crowders can alter the physicochemical properties of BMCs, including their interfacial energy, viscosity, and internal organization, thereby affecting the dynamics of biomolecular processes that take place inside condensates or that are regulated by BMCs. Modulation of BMC interfacial energy and viscosity influences the kinetics of the phase separation process, including nucleation, growth and coarsening, size and size distribution of BMCs. Therefore, it can be asserted that the interplay between crowding and LLPS can ultimately govern the formation and evolution of membraneless organelles, adding a further layer of complexity (Figure 6).

The molecular driving forces underlying the formation, properties and dynamics of BMCs are often investigated *in vitro* using reconstituted components, or model systems that mimic BMC properties. Among these are coacervates that are small droplets formed by LLPS.^283^ Coacervates can be prepared from a wide range of biomolecules, including peptides, proteins, RNA, polysaccharides and small molecules, and synthetic polymers.^284^ Over time, these coacervate microdroplets undergo coalescence, ultimately resulting in the formation of a dense bulk phase (the bulk coacervate).

The process of coacervation is described either as simple (homotypic) or complex (heterotypic). Simple coacervation occurs through the self-association of proteins upon changes to the environmental conditions, such as changes in temperature and pH,^285,286^ and is usually associated with hydrophobic forces between solvent-exposed residues. Classical examples are the phase separation of histidine-rich peptides inspired by disordered squid beak proteins, HBPs,^287^ and arginine/tyrosine-rich peptides inspired by mussel-foot proteins, MFPs (*vide infra*).^288,289^ Complex coacervation arises from the encounter between macromolecules possessing opposite charges or other forms of complementary interactions, and is favored by the presence of proteins with nucleic acid-binding domains, low complexity domains,^290−292^ and RNA molecules with protein binding sites.^293^ A well-known example of such interactions occurs between positively charged proteins, like histones, and negatively charged nucleic acids, which can give rise to LLPS *in vitro* and *in vivo*, pointing to a role in chromatin organization.^294,295^ Other notable examples involve biomacromolecules such as RNA with short cationic peptides or two oppositely charged proteins.^296,297^

Different forms of coacervates play a role in different biological processes, as for instance cellular compartmentalization, cell replication, and vesicle formation, all promoted by highly crowded environments. The most well-known membraneless organelles are the nucleoli inside the nucleus,^282^ Cajal bodies, which are involved in the assembly of small nuclear ribonucleoprotein and in ribosome biogenesis,^298^ stress granules which are formed under stress conditions,^281^ and RNA granules, which are involved in the transport and delivery of crucial cellular components to far-away parts of neurons.^299,300^ Besides their importance in biology and owing to their extremely variable composition and topology, coacervates also play a crucial role in man-created fields, such as food industry,^301^ biophysics, biomaterials,^302,303^ and drug delivery.^304^

### 6.4 Role of Crowding as Inducer and Promotor of LLPS

Access to an understanding of the physicochemical foundations of LLPS is provided by the Flory–Huggins theory^50−53^ established in the forties of the last century (Section 2.5). The impact of crowding on LLPS is a complex and not yet fully understood combination of factors. One of the primary effects of crowding on LLPS is its effect on the phase diagrams of proteins and other phase-separating molecules, usually resulting in crowding agents inducing or promoting LLPS (Figure 6). Indeed, synthetic crowding agents, such as PEG, Ficoll or dextran, are commonly added in studies of protein LLPS. André and Spruijt summarized the use of these crowding agents in cell-free studies of LLPS.^305^ In the cases where comparative studies without crowding were available, the addition of crowding agents was found to induce or promote phase separation, evidenced by a decrease in the protein concentration required for phase separation or the absence of droplets without crowding agents. Well-known examples include tau, G3BP1, hnRNPA1, FUS, and NPM1/RNA. Park et al. also found that crowding by PEG significantly increases the volume of coacervate formed by polylysine and hyaluronic acid, which was attributed to a dehydration of the polymers by PEG that leads to a strengthening of their interaction.^306^ However, in most cases, the effect of crowding was not systematically investigated. Moreover, detailed investigations into the mechanism and strength of the effect of crowding on LLPS, and how the molecular characteristics of the crowding agents (chemical structure, molecular weight) are scarce. Both the chemical structure of the crowding agents, and their size (with respect to the protein) have been shown to influence the effect that crowding has on LLPS in specific cases.^307,308^

The general picture emerging from studies of LLPS *in vitro* and *in vivo* is that crowding tends to induce or promote phase separation, both for simple and complex coacervates, and both for disordered proteins and modular proteins with folded domains. Three mechanisms have been proposed to underlie the LLPS-promoting effect of crowding. From a colloid science perspective, depletion interactions are known to become stronger with increasing crowder concentration, and the interaction range increases with increasing the relative size of the crowder. Mapped onto phase separation of disordered proteins, nonspecific interactions of “inert” crowding agents could increase the attraction between proteins as a result of enhanced depletion.^309^ Globular proteins, such as BSA and HSA are known to undergo LLPS into coacervate droplets in the presence of PEG.^310,311^ A compaction of several IDPs was also observed in the presence of crowding agents, the effect being dependent on crowder size and quantitatively coherent with the theory of depletion interactions when considering the IDPs and crowders as polymers.^312,313^ Whether depletion-induced attraction alone is sufficient to induce phase separation of IDPs remains to be seen: the increase in attraction between disordered proteins caused by crowding agents acting as depletants is expected to be less pronounced than between globular proteins.

If we then adopt the perspective of polymer science, segregative and associative interactions between different polymers are known to result in LLPS.^314^ Well-known examples include the segregation between PEG and dextran in two coexisting liquid phases and the hydrogen bond-mediated association between PEG and poly(acrylic acid) at low pH, resulting in associative phase separation. Analogously, polymeric crowding agents and disordered proteins and nucleic acids may exhibit either segregative or associative interactions that can lead to phase separation. However, quantification of these intermolecular interactions requires carefully planned experiments, while qualitative indicators such as the appearance of the phase diagram may be misleading. As an example, the phase diagram of the protein FUS and PEG qualitatively resembles the phase diagrams of polymers that are known to undergo segregative phase separation.^315,316^ In this case, a depletion of the crowding agent from the coacervates or condensates is expected. However, tie line analysis revealed that FUS exhibits an attractive interaction with PEG and PEG is strongly concentrated in the condensates.^317^ In another study by Lemetti et al., the Gln/Ala-rich disordered silk-like triblock protein CBM-eADF3-CBM was found to undergo LLPS more easily in the presence of dextran.^318^ The experimental phase diagram also in this case resembled segregative phase separation, and indeed dextran was found to be excluded from the coacervates. Ficoll was found to induce phase separation of equimolar SH3_5_-PRM_5_ mixtures in a segregative manner, according to the phase diagram, and was again excluded from the condensates.^319^ PEG was found to be excluded from coacervates of spermine/polyU studied by Marianelli et al.,^307^ and from coacervates of tau/polyA studied by Hochmair et al.^320^

On the other hand, the phase diagram of G3BP1, a key component of stress granules, and polyA RNA resembles diagrams of associative phase separation. For this case, an enhanced concentration of the crowding agents in the coacervates is expected, an effect that has indeed been observed for dextran in coacervates formed by the RGG domain of LAF-1.^308^

Several recent studies have further investigated the effect of crowding on LLPS in more details, pointing out that the addition of neutral crowders can significantly influence the occurrence of phase separation, the size and size distribution of liquid droplets, as well as the kinetic path of phase separation. Bai et al.^321^ investigated the influence of macromolecular crowding on LLPS of oppositely charged polyelectrolytes with an arginine-rich block and a single-stranded oligonucleotide, and found that the presence of crowders enhanced the nucleation and growth, via excluded volume effects. However, crowding also suppressed Brownian-motion-based coalescence, effectively trapping the coacervate droplets in the crowder network. As a consequence, the size of the coacervate droplets decreased linearly with increasing crowder concentration, the effect being stronger for PEG than for polyacrylamide. Interestingly, the authors also found that PEG was localized inside the coacervates, resulting in changes in the stability and dynamics of the formed droplets, as we will discuss in more detail in the following section.

André et al. recently investigated the influence of PEG as a macromolecular crowder on the phase separation behavior of NPM1 and rRNA.^322^ NPM1 can bind to RNA with relatively weak multivalent interactions, driving LLPS. The authors determined part of the phase diagram and found that the binodal was shifted in the presence of PEG, with the effect being stronger for NPM1. NPM1 could phase separate also in the absence of RNA but in the presence of PEG, whereas did not phase separate in the absence of RNA and PEG. Experiments with labeled PEG revealed that, in this case, the crowding agent is concentrated in the condensates, suggesting that an associative interaction between PEG and NPM1 underlies the crowding-induced and crowding-promoted phase separation.

Delarue et al. showed that ribosomes can act as crowding agents that enhance phase separation of a homodecamer repeat of SUMO and a homohexamer SUMO interaction motif (SIM) both *in vitro* and *in vivo*.^323^ They looked at the probability of finding SUMO:SIM condensates in cells with different ribosome concentrations, treated with rapamycin in yeast deletion strains that had previously been determined to affect crowding, and found a strong correlation between ribosome concentration and probability of finding phase separated SUMO:SIM inclusions. The authors found that *in vitro* the local concentration of SUMO and SIM in phase separated droplets was 50% higher when adding ribosomes as crowders, purified from *E. coli* to a level that resembles the *in vivo* conditions, indicating that crowding also influences the composition and, probably density and viscosity of the condensates (*vide infra*).

To better quantify the influence of crowding on LLPS of proteins, measurement of the phase diagram is invaluable. However, practical factors, including the availability of purified proteins, often pose limitations to the mapping of protein phase diagrams with high resolution. To circumvent the problem, Arter et al. recently presented a combinatorial microdroplet platform to measure the phase diagrams of disordered proteins, among others.^316^ The authors showed that their platform allows determination of how small molecules and crowding agents modulate the phase diagram by calculating a differential phase diagram in the absence and presence of the additives. This method holds great promise for studies of crowding on protein LLPS.

### 6.5 Crowding and Biomolecular Condensate Physical Properties

Crowding does not only promote LLPS, but it can also alter the composition and fundamental physicochemical properties of condensates, in contrast to the case of protein aggregation (Figure 6). Several recent studies have shown effects of crowding on coacervate or condensate composition, viscosity, surface tension, and droplet size.

The above cited André et al., for instance, found that addition of PEG changes the composition of NPM1/rRNA condensates: while the local concentration of NPM1 increased 3-fold upon addition of 2% PEG, the local concentration of RNA remained practically unchanged.^322^ At the same time, PEG was concentrated inside the condensates, indicating that not only the composition of the condensate had changed with crowding, but also the density. Further studies using fluorescence recovery after photobleaching (FRAP) revealed that both the dynamics of recovery of NPM1 and RNA was slowed down by crowding. At low crowder concentration both NPM1 and RNA displayed partial recovery, suggesting that the effective viscosity of the condensates had increased because of an increase in attraction between the components. However, at higher crowder concentration, no recovery was observed, indicating that the condensates had lost their fluid nature and became gel-like. Ferrolino and co-workers also found that the mobility of NPM1 of homotypic droplets decreased rapidly with crowding.^324^ Experimental observations by Hochmair et al. on tau/polyA coacervates also indicated that crowding-induced coacervates have a higher density than coacervates formed under noncrowded conditions.^320^ They found that binary tau:polyA coacervates and binary, noncrowded coacervates of tau with other polyanions (heparin, tRNA, polyU), could not be pelleted by centrifugation, while PEG-induced tau/polyA coacervates could be pelleted under the same conditions, suggesting that noncrowded coacervates have a similar density to the surrounding aqueous solution, and a lower density than PEG-induced coacervates. In contrast, Lemetti et al. found that crowding by dextran did not increase the effective viscosity (and, by extension, density) of silk-like protein coacervates, as the fluorescence recovery after photobleaching occurred on the same time scale in the absence and presence of dextran.^318^

Jo and co-workers recently studied the recruitment of various client proteins (IDPs with different sequence composition) into condensates formed by either LAF or FUS-derived IDPs.^325^ They found that increasing crowder concentration increased the partitioning of almost all client IDPs. The effect was observed for all crowders tested (PEG, BSA, dextran), but it was significantly more pronounced for PEG than for any of the other crowders. Some partition coefficients increased nearly an order of magnitude upon increasing crowder concentration from 5 to 15%. The presence of tyrosine and arginine residues in the client proteins contributed strongly to the recruitment of the clients. Interestingly, PEG was also the only crowder that itself was found to be concentrated in the condensates.

Bai and co-workers investigated how the presence of crowders in the solvent or their participation in phase separation varies the interfacial energy of the droplet.^326^ In the case of multiphase coacervates, changes in the interfacial energy could tune the morphology, generating attractive hierarchical structures. To test this hypothesis, the authors studied the coacervation of poly(l-lysine) (PLL), quaternized dextran (Q-dextran), and ss-oligo in crowded media provided by PEO or dextran. The authors prepared solutions of PLL/Q-dextran and ss-oligo separately, which were later mixed to form biphasic coacervates. Without crowders, PLL, Q-dextran, and ss-oligo spontaneously formed biphasic coacervate droplets with specific arrangements of internal and external phases. However, the introduction of PEO at varying concentrations led to intriguing transformations. At lower PEO concentrations, droplets connected and eventually formed a giant PLL/ss-oligo core surrounded by Q-dextran/ss-oligo droplets. As PEO concentration increased, the droplets evolved into complex structures, including branched and networked patterns. Interestingly, dextran exhibited distinct effects from PEO. Droplets maintained their original shape and structure in dextran solutions, even at high concentrations. This behavior could be attributed to the compatibility of dextran with the components of the coacervate. The addition of PEO to dextran-containing solutions resulted in rapid merging of particles, forming large droplets.

Shillcock and co-workers investigated the influence of crowding on condensates modeled on the IDP FUS using dissipative particle dynamics simulations.^327^ Their computing framework allowed dozens of simultaneous simulations spanning the protein/crowder concentration space to search the high-dimensional parameter space and rapidly locate regions of interest to make experimentally relevant predictions. Their results confirmed that crowding can enhance phase separation, with the steric repulsion by the crowding agent driving a system across the phase boundary. However, the resulting condensates were insensitive to the crowder concentration: similar composition and density were found for crowded and noncrowded condensates, suggesting that also the viscosity remains the same. It is not clear if the results would be different for crowders that do exhibit specific interactions with the phase separating proteins apart from steric repulsion, such as PEG.

Crowding can also impact another biologically important property of condensates or coacervates: their size (Figure 6). Thermodynamics predicts that the formations resulting from the nucleation of small coacervates will coarsen through coalescence and ripening of the droplets to ultimately form a single bulky coacervate. However, cells typically contain a distribution of separate condensates of a seemingly well-defined size. The fact that certain cellular condensates seem to resist coarsening and remain stable at a given size has been attributed by some to an active formation process.^328^ However, it has recently been shown that metastability can also arise from an interplay between two dynamic processes: diffusion-limited encounter between proteins and the exhaustion of available valences in smaller clusters,^329^ or between nucleation and coalescence.^330^

Crowding can alter all these processes: coarsening of coacervates is driven by a decrease in interfacial energy, and requires diffusion and collision between coacervate droplets. As discussed above, crowding has been shown to alter the interfacial energy of coacervates, with increased crowding decreasing the interfacial tension of typical condensates, an effect that is in agreement with other reports on multiphase coacervates.^331^ In addition, a crowded environment also decreases the collision frequency between colloidal particles, including emulsion droplets, undergoing Brownian motion, as observed by Bai et al.^321^ Both effects will suppress the coarsening of coacervates, possibly down to levels where micron-sized condensates appear to be metastable. Consequently, the crowded state of the cell may constitute an additional mechanism through which condensate size is regulated.

Vweza et al. used computational modeling to investigate the effect of crowding by ribosomes on condensate coarsening.^332^ They extended the conventional Cahn–Hilliard model with experimentally derived macromolecular crowding dynamics and state-dependent reaction kinetics and showed that crowding results in smaller droplets that were coarsened at a late stage of the evolution of the field, while further increasing the crowder concentration resulted in labyrinthine patterns that did not relax to round droplets, reminiscent of arrested gel-like phases. Such arrested gel-like phases at high crowding have previously been found in coacervates, as discussed above, although the gel-like coacervates always appeared as spherical droplets.

Experimental observations of the influence of crowding on the size of coacervates are scarce. Moreover, these results may be convoluted with an effect of crowding on the lowering of the critical protein concentration required for phase separation. For example, Fang et al. found that FCA, a floral repressor protein, forms only very small condensates in the absence of the crowding agent PEG, whereas the addition of PEG resulted in a significant increase in condensate size and number.^333^ Similarly, in the above-mentioned studies, André et al. found that condensates formed by NPM1-rRNA were small in the absence of PEG, and significantly larger when PEG was added. In both cases, the effect was attributed to a promotion of LLPS by PEG.

These findings have broad implications for our understanding of the formation of membraneless organelles and behavior within crowded cellular environments. Different groups have highlighted how the dynamic interaction between macromolecular crowders and coacervate components can dictate the structure and function of membraneless organelles. The cellular interior comprises diverse molecules, each with potential affinities for specific phases or subphases of the organelles. Therefore, the crowded environment may act as a regulatory mechanism, responding to external changes and influencing the cellular organization and functionality.

### 6.6 Crowding and the Links between LLPS and Aggregation

Notably, aberrant phase separation can lead to protein aggregation and the formation of pathological aggregates, implicated in neurodegenerative diseases such as Alzheimer’s, Parkinson’s, and amyotrophic lateral sclerosis (ALS). The crowded cellular environment and its influence on phase separation dynamics may contribute to the aggregation propensity of disease-associated proteins. Therefore, investigating the interplay between macromolecular crowding, LLPS, and protein aggregation can provide insights into the molecular mechanisms underlying neurodegeneration and potentially lead to therapeutic strategies. In this context, Hochmair et al. investigated how tau protein condensates contribute to disease-associated cellular tau accumulations.^320^ Tau is an intrinsically disordered protein which plays a crucial role as a neuronal microtubule binding protein, contributing to the stability of axons in the central nervous system. Interestingly, tau exhibits diverse assembly forms, each with unique biophysical and biochemical properties that dictate its cellular functions. Monomers and dimers are considered to resemble the soluble “native” tau form in the cytosol. Tau oligomers are implicated in neurotoxicity and have been associated with the seeding of aggregation and spread of tau pathology between neurons. β-structured tau aggregates are the stable end products of aggregation, accumulating in long-lasting neuronal inclusions. Additionally, liquid-like condensates of tau, formed through LLPS, have emerged as essential for various tau functions, including microtubule binding, polymerization, and formation of seeding-competent tau oligomers. *In vitro* studies have identified two modes of tau condensation: crowding-induced LLPS,^334,335^ and complex coacervation.^336−338^ Crowding agents like PEG or dextran induce tau demixing into liquid-dense condensates. These condensates are proposed to harbor pathological seeding potential and can convert into oligomeric tau species similar to *in vitro* aggregates. On the other hand, complex coacervation occurs when tau co-condensates with polyanionic RNA, forming liquid-like droplets through electrostatic interactions.

To understand how different types of biomolecular tau condensates contribute to tau biology and disease-associated cellular tau accumulations, Hochmair and co-workers conducted a comprehensive study to characterize tau condensation under physiologically relevant conditions *in vitro* and its functional roles in the context of microtubule binding, polymerization, and pathological aggregation.^320^ Results revealed that molecular crowding plays a critical role in enabling the condensation of tau and phospho-tau. Specifically, the authors found that at physiological cytoplasmic ion concentrations, molecular crowding is essential to facilitate the condensation of both tau and phospho-tau. In the absence of crowding, tau phosphorylated at specific sites is unable to coacervate with RNA. This finding underscores the importance of considering the interplay between molecular crowding, post-translational modifications, and other biomolecules in driving the formation of liquid-like dense tau phases. Regarding the pathophysiological potential of tau condensates, the study deviates from the typical progression of biomolecular condensates into aggregates. Unlike other proteins,^311^ tau condensates do not necessarily percolate into aggregates and instead, remain in a condensed phase.

Also in other systems, condensates do not always transform into percolated fibrillar networks upon aging, but the condensate state does change upon aging. This change in material state can be influenced by crowding. Kaur and co-workers studied the solidification of FUS in the presence of crowding by PEG.^315^ FUS and FUS mutants have previously been found to transform into fibrillar structures upon aging *in vitro*.^339^ However, in the presence of PEG, FUS condensates do not change their spherical morphology upon aging, but they lose the ability to fuse or recover from photobleaching.^315^ Full-length FUS transits from a viscous fluid state to a viscoelastic gel-like state gradually in a crowding-dependent manner. This gradual effect was also observed for the FUS RGG domain, but not for the FUS prion-like domain (FUS-PrLD). FUS-PrLD switched more abruptly to an arrested gel-like state at 15% PEG. This effect was independent of the molecular weight of PEG and dextran and was attributed to the general increase in intermolecular interactions caused by volume exclusion.

These examples demonstrate that phase transitions in the cell can be both functional and adverse and must be carefully controlled for correct cellular functioning. Crowding may affect these phase transitions at many different levels, and can ultimately govern phase separation and aggregation, adding a further layer of complexity. Studying the effects of crowding on aggregation and LLPS not only brings new insights into crowding itself, but it also deepens our understanding of the properties and dynamics of aggregates and biomolecular condensates.

## 7 A Case Study from Nature: Exploring the Impact of Molecular Crowding on Mussel Foot Proteins

In the following sections, we will discuss a specific example where an interplay between LLPS, aggregation and crowding takes place. The choice of the system is arbitrary, but well exemplifies the different aspects discussed in this chapter and how, despite the large plethora of studies on crowding, much still needs to be tackled. The system is also a bit exotic and very interesting, as it involves a marine organism well-known to all of us: mussels. Much could be studied to elucidate the effect of crowding on mussel-inspiered bioadhesives, which have captured interest for their potential applications in various biomedical fields, including regenerative medicine, tissue engineering, surgery, and implantation of medical devices.

### 7.1 How Mussels Attach to Wet Surfaces

**Figure 7:**
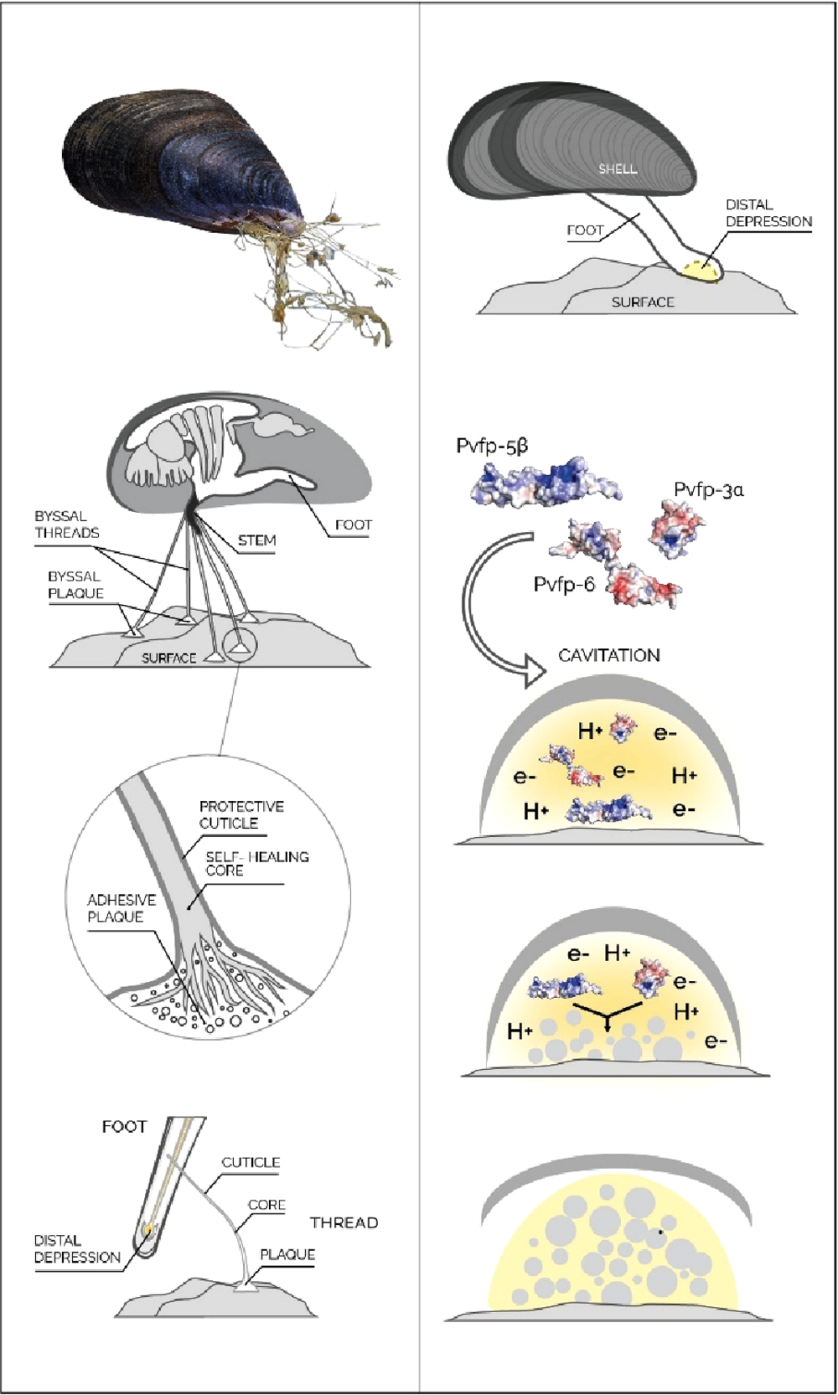
Mussel byssal plaque’s formation and deposition. On the left, from the top to the bottom: Mussel generates several byssal threads ending with plaques firmly attached to the surface. Below follows schematic anatomic representation of byssal threads, from the stem where the threads’ elongation starts, to the core formed inside the elongated thread, protected by a cuticle and the adhesive plaque. On the right, from the top to the bottom: schematic representation of the different steps for the plaque formation and deposition. Mussel foot anchors on the surface and creates a cavitation, known also as distal depression, which allows to maintain different chemical conditions than those of the outer environment. Under acidic pH, low ionic strength and controlled redox conditions, the mussel foot proteins responsible of the adhesion process (Pvfp-5β, Pvfp3α, and Pvfp-6) are secreted and undergo coacervation to enhance spreading and wettability on the surface. Finally, immediately after the foot is released the plaque undergoes solidification in contact with seawater and firmly remains anchored to the surface.

Mussels have evolved a remarkable adaptation to survive in the dynamic and challenging coastal environment they inhabit. Central to their survival is their exceptional ability to firmly attach to various wet surfaces using specialized proteins, commonly referred to as Mussel Foot Proteins (MFPs).^340,341^ MFPs play a crucial role in the formation of the mussel byssal plaque, a porous and fibrous adhesive structure that enables mussels to anchor securely, resist the relentless forces of waves and currents, and maintain their position on diverse substrates such as rocks, ship hulls, or other organisms.^340,342−347^ Several MFPs have been identified in the different mussel gena. In the Asian green mussel *Perna viridis* for instance there are three (Pvfp-3α, -5β, and -6), which are secreted with a well-defined temporal succession.^342^ To adhere on the desired surface, the mussel first employs its foot to create a specialized and isolated reaction chamber (cavitation) with specific conditions which differ from those of the surrounding seawater environment, including low pH, low ionic strength, and highly reducing poise (Figure 7). These unique conditions facilitate a controlled process that involves LLPS, surface adsorption, spreading, formation of microstructures, and ultimately, solidification of the adhesive proteins into the byssus. The orchestrated sequence of these events allows precise deposition and formation of a durable adhesive material (Figure 7).

MFPs have captured scientists’ interest for decades, because of their exceptional adhesive strength, that surpasses many synthetic adhesives, and the impressive resistance to harsh environmental conditions, including high-salt seawater, fluctuating temperatures, and mechanical stress.^340,348^ MFPs are also biocompatible, making them attractive for their potential applications in various biomedical fields, including regenerative medicine, tissue engineering, surgery, and implantation of medical devices.^349−357^

The adhesive properties of MFPs can be attributed to their unique sequence composition and structure. The presence of specific amino acids and post-translational modifications enhances their durability, enabling mussels to maintain their attachments for extended periods. A crucial role in the adhesive process is played by the catecholic amino acid 3,4-dihydroxyphenylalanine (DOPA), derived from post-translational modification of tyrosine. DOPA residues, of which MFPs are rich, facilitate strong and versatile interactions with various molecules, including metal oxides, minerals, and organic polymers.^358−363^ The presence of other amino acids, such as lysines and arginines, has been recently recognized as another main contribution to the overall adhesive performance of MFPs.^341,363−367^ DOPA and positively charged residues (i.e., Lys and Arg) exhibit considerable spatial correlation in MFPs, promoted by cation-π and hydrophobic interactions. DOPA can reduce the stability of the hydration layer on the surface, eliminate the hindrance caused by ions, and consequently facilitate the binding of basic residues to the negatively charged marine surface by electrostatic interactions, which contribute substantially to the binding stability of MFPs.^363,365,366^ Tyrosine residues can also form interactions with positively charged basic residues suggesting that the post-translational modification of Tyr to DOPA is not necessarily needed to explain the adhesive properties.^350,366,368^

Notably, MFPs possess a unique hierarchical structure that further enhances their adhesive properties: most MFPs consist of repeats of β-rich domains.^342,350,369−371^ Although the experimental three-dimensional structure is available only for Pvfp-5β from the Asian green mussel *Perna viridis* (PDB ID 7QAB),^371^ computational predictions have suggested that MFP domains are interconnected by flexible linkers, allowing the proteins to undergo conformational changes and adapt to different surface topographies. Hierarchical assembly of MFPs involves first the formation of condensates that suddenly evolve in nanoscale structures, such as amyloid-like fibers,^371−374^ which contribute to the bulk adhesive properties of the byssus.

To fully harness the potential of MFPs and optimize their use in various applications, it is crucial to understand the influence of environmental factors on these proteins, including the effect of molecular crowding which has been so far overlooked. LLPS and coacervation of MFPs have gained great interest,^362,371,372,374−376^ whereas, to our knowledge, there is only one specific study that mentions the effect of self-crowding on MFPs.^377^ This is particularly peculiar since the copresence of the different MFPs must naturally induce crowding during the process that leads to the formation of the adhesive byssal plaque. Moreover, as well described in the previous section, crowders can tune condensates kinetics, composition, density, viscosity, size, and surface tension. In the finding of new mussel-inspired biomaterials, it is therefore crucial to know how it is possible to tune the adhesive proprieties of the system by the use of molecular crowders.

### 7.2 LLPS in MFPs’ Adhesion

While the effect of molecular crowding on the MFP properties has been severely overlooked, several investigations have provided valuable insights on the LLPS process in MFPs adhesion.^362,371,372,374,376,378^ These studies have stressed, albeit indirectly, the importance of crowding on the MFP functions.^144,379−384^ Upon acidification of the distal depression (the reaction chamber created by the mussel foot), MFPs are swiftly secreted, and a series of events unfold involving both their adsorption onto surfaces as solutes and their condensation through LLPS.^385^ Notably, coacervation of mussel adhesive proteins is described by the involvement of single components rather than paired oppositely charged molecules, and is not necessarily charge neutral, with H-bonding, cation−π and π–π interactions being responsible for LLPS (cohesive interactions).^362,371,373,385,386^ The condensation process of MFPs is carefully regulated by the transition from acidic to basic pH conditions and the change of ionic strength.

Coacervation is extremely important for MFPs’ underwater adhesion. Indeed, coacervates are denser than water and so can directly adhere to a surface without being diluted by diffusion. They also possess low interfacial energies, enabling them to spread over wet surfaces and protect against unfavorable chemical processes such as DOPA oxidation.^375,387^ DOPA contributes to byssal plaque adhesion, but only if protected from oxidation at the interface with the marine surface. This condition is met since, although DOPA oxidation to DOPA-quinone is thermodynamically favorable in seawater, DOPA-quinone is almost absent in the byssal plaques and the interfaces between plaques and substrate remain reduced for months.^388^ This is possible thanks to coacervation that serves as a natural mechanism to safeguard against oxidation.^389,390^ Through coacervation, oxidation-prone groups can be sequestered within fluid-filled inclusions situated in the porous structure of the byssal plaque. This strategic arrangement effectively shields the groups and allows them to participate in redox reactions that would instead be impaired by oxidation.^375^

Although significant knowledge exists regarding the chemistry behind plaque adhesion, a considerable gap remains in our understanding of the process by which plaques are formed. A recent investigation by Renner-Rao et al.^374^ utilized advanced 3D electron microscopic imaging techniques to delve into the structure and formation of mussel byssal plaques. Intriguingly, their findings shed light on the spontaneous development of micro- and nanopores during the secretion of vesicles filled with proteins. Within each vesicle, a sulfate-associated fluid condensate containing proteins enriched with DOPA was observed. Notably, when these vesicles were broken under specific buffering conditions, a controlled multiphase LLPS occurred which involved the separation of different proteins. This led to the formation of a continuous phase coexisting with droplets. Cross-linking of the continuous phase by pH modification resulted in the generation of solid porous structures, referred to as microplaques, with the droplet proteins remained as fluid condensates confined within the pores. These findings offer intriguing insights, suggesting that the combination of phase separation and the ability to modulate cross-linking could serve as an effective strategy for fabricating hierarchically porous materials via self-assembly. Interestingly, it was demonstrated that the granular substructures responsible for the formation of the mussel cuticle (a specialized outer layer or coating found byssus, Figure 7) are also prearranged within condensed liquid phase secretory vesicles. These vesicles exhibit phase separation, and during secretion their components fuse together, forming the substructure of the cuticle.^372^

In conclusion, LLPS is a remarkable aspect of the dynamic nature of MFPs. Liquid condensates can undergo fusion, fission, and coalescence, allowing rapid remodeling and adaptation of the adhesive material. Consequently, the reversible nature of phase separation allows dissipation of mechanical stress and formation of adhesive contacts, making the adhesive material resilient and adaptable to different conditions. However, despite the progress made in understanding LLPS in MFPs, several questions and challenges remain open, including the specific role of molecular crowding.

### 7.3 How Crowding Could Affect the MFPs’ Behavior

Molecular crowding can modulate the folding, stability, and overall conformational landscape of MFPs, leading to significant changes in their adhesive properties. More importantly, crowding can affect LLPS processes involving MFPs by altering the condensate composition, size, density, viscosity, and surface tension (see section 6.5).

In crowded environments, the increased concentration of macromolecules can lead to excluded volume effects, which restrict the conformational space available for protein self-interaction and coacervates formation. Conversely, crowding can also increase the propensity for non-native interactions and aggregation, potentially destabilizing the folded state of MFPs and leading changes in the condensate features. On the other hand, crowding can influence the stability of MFPs by modulating their interactions with surrounding molecules.^133,141−147,391^ This can result either in an increased protein stability or in protein destabilization and loss of functionality.

Crowding could also modulate the conformational dynamics of MFPs by affecting their internal motions and flexibility.^137−141^ An excess of crowders can restrict the movement of MFPs, reducing their conformational entropy and promoting more ordered conformations. This restriction in conformational space can have implications for the adhesive function of MFPs, as it can impact their ability to undergo structural rearrangements required for effective adhesion.

MFPs aggregation can also be significantly impacted by crowding, and this in turn would influence both the formation of the byssal treads and plaques and their adhesive properties. Indeed, crowding can promote or suppress protein aggregation, depending on various factors such as protein concentration, crowding agent properties, and environmental conditions (see section 6.1).^69,163,392,393^ Modulation of crowding-induced aggregation of MFPs can lead either to the formation of larger supramolecular assemblies or to prevention of aggregation by stabilizing the soluble state of the proteins through excluded volume effects and intermolecular interactions. Consequently, controlled aggregation of MFPs can be harnessed to develop materials with tunable adhesive strength and toughness.

The copresence of several MFPs together in the confined space of the distal depression can also promote the formation of additional intermolecular contacts between MFPs and surfaces, and/or of intermolecular cross-links and supramolecular assemblies. These additional interactions can enhance adhesion by providing stronger binding forces and increasing the contact area at the interface. Moreover, the formation of higher-order structures, such as oligomers or aggregates, can provide additional mechanical stability and toughness to the adhesive interface. On the other hand, crowding can also lead to increased steric hindrance and competition for binding sites, potentially affecting the accessibility of key adhesive motifs in MFPs.

Interestingly, a recent study by Lu et al. has shown that coacervate-membrane interactions are mainly governed by the coacervate surface properties, resulting in different wetting morphologies.^394^ Similarly, tau condensates nucleating preferentially on microtubule filaments *in vivo* can be explained by a wetting transition.^395^ Crowding can alter the interfacial tension of coacervates, and thereby also their interaction with other biomolecular structures such as cell membranes and filaments. These results are particularly important for the application of mussel-inspired bioadhesives in regenerative medicine, where tissues healing and function restoration relies on cell–cell adhesion. However, this aspect of crowding has not been investigated in detail. Some experimental observations suggest that crowding indeed influences coacervate interaction with surfaces. tau:polyA coacervates were found to wet negatively charged surfaces (glass) more readily in the presence of PEG as a crowding agent,^320^ suggesting that crowding could also lead to better wetting and bundling of tau:RNA-based condensates on negatively charged microtubules. It would be interesting at this point to investigate the potential of crowding agents to tune the interaction of MFPs condensates with cellular membranes and, more in general, with other surfaces.

## 8 Conclusions

In summary, we have discussed here how the concept of molecular crowding has evolved from its first conception and how the structure and dynamics of biomolecules in cell-mimicking environments have increasingly gained the attention of researchers. It is clear that we have come a long way from the first studies based on polymer physics to reach a much more realistic model of the cellular environment. We have discussed the value of various environment conditions and evaluated the use of different crowders. This review may hopefully offer a comprehensive, even though certainly incomplete, description of the effects of crowding on different cellular processes such as protein structure, aggregation, and phase transitions. We hope that our work might serve as a valuable guideline for the future design of new approaches to the study of molecular crowding. Finally, we have discussed at some length the specific example of MFPs. The choice was dictated by two intertwined considerations. The first was to present a case in which the potential influence of crowding is clear. At the same time, the potentialities of this system as a biomaterial impose that we understand well how the addition of crowders may modulate its properties. Investigating the impact of crowding on the adhesive strength of these proteins will however require sophisticated experimental approaches that can accurately replicate the crowded conditions found in natural mussel habitats. Careful selection of appropriate crowding agents and their concentrations will be crucial for mimicking the natural crowded environment in experimental setups.
